# Microenvironment modulation by key regulators of RNA N6-methyladenosine modification in respiratory allergic diseases

**DOI:** 10.1186/s12890-023-02499-0

**Published:** 2023-06-16

**Authors:** Yuting Wang, Jiaxi Wang, Zhanfeng Yan, Siming Liu, Wenlong Xu

**Affiliations:** 1 Department of Otorhinolaryngology, Dongfang Hospital Affiliated to Beijing University of Chinese Medicine, Beijing, China; 2 Department of Otorhinolaryngology, Dongzhimen Hospital Affiliated to Beijing University of Chinese Medicine, Beijing, China

**Keywords:** m6A regulators, m6A modification, Immune microenvironment, METTL14, Respiratory allergic diseases, Allergic rhinitis, Asthma

## Abstract

**Background:**

RNA N6-methyladenosine (m6A) regulators are considered post-transcriptional regulators that affect several biological functions, and their role in immunity, in particular, is emerging. However, the role of m6A regulators in respiratory allergic diseases remains unclear. Therefore, we aimed to investigate the role of key m6A regulators in mediating respiratory allergic diseases and immune microenvironment infiltration characteristics.

**Methods:**

We downloaded gene expression profiles of respiratory allergies from the Gene Expression Omnibus (GEO) database and we performed hierarchical clustering, difference analysis, and construction of predictive models to identify hub m6A regulators that affect respiratory allergies. Next, we investigate the underlying biological mechanisms of key m6A regulators by performing PPI network analysis, functional enrichment analysis, and immune microenvironment infiltration analysis. In addition, we performed a drug sensitivity analysis on the key m6A regulator, hoping to be able to provide some implications for clinical medication.

**Results:**

In this study, we identified four hub m6A regulators that affect the respiratory allergy and investigated the underlying biological mechanisms. In addition, studies on the characteristics of immune microenvironment infiltration revealed that the expression of *METTL14*, *METTL16*, and *RBM15B* correlated with the infiltration of the mast and Th2 cells in respiratory allergy, and *METTL16* expression was found to be significantly negatively correlated with macrophages for the first time (R = -0.53, *P* < 0.01). Finally, a key m6A regulator, *METTL14*, was screened by combining multiple algorithms. In addition, by performing a drug sensitivity analysis on *METTL14,* we hypothesized that it may play an important role in the improvement of allergic symptoms in the upper and lower airways with topical nasal glucocorticoids.

**Conclusions:**

Our findings suggest that m6A regulators, particularly *METTL14*, play a crucial role in the development of respiratory allergic diseases and the infiltration of immune cells. These results may provide insight into the mechanism of action of methylprednisolone in treating respiratory allergic diseases.

**Supplementary Information:**

The online version contains supplementary material available at 10.1186/s12890-023-02499-0.

## Introduction

Respiratory allergies include asthma and allergic rhinitis (AR), which are the most common chronic inflammatory diseases of the respiratory tract worldwide with marked heterogeneity and complex pathophysiological manifestations. Asthma and AR can severely affect patients' social life, quality of life and are associated with sleep disturbances and negative emotions [1]. With the recent increase in ambient air pollution, the incidence of AR and asthma is as high as 31.6% and 2.4%, respectively [2]. Studies showing similar physiological reactions to various stimuli in patients with asthma and AR have led to a unified airway theory [3–6]. Structurally, the mucosa from the nasal cavity and lower respiratory tract is highly similar. Therapeutically, systemic therapeutic approaches (e.g., immunotherapy) tend to produce better outcomes for the control of upper and lower respiratory tract allergic diseases [7]. While there is agreement on the theory of the unified airway, further research is necessary to fully understand the development, progression, and treatment of diseases affecting both the upper and lower airways.

In addition, respiratory allergies are thought to be caused by environmental factors acting on genetically susceptible individuals and are regulated by epigenetics [8]. RNA N6-methyladenosine (m6A) modification is an epigenetic modification, and m6A regulator-mediated RNA methylation is considered to have diagnostic, therapeutic, and prognostic potential in immune system diseases [9]. m6A modification is a methylated modification that occurs on the N atom at position 6 of RNA adenine (A) [10]. m6A modification is performed by m6A methyltransferases (“writers”), removed by m6A demethylases (“erasers”), and perceived by m6A-specific binding proteins (“readers”). Currently, there are some studies on the regulation of respiratory allergies by m6A. First, a study has shown the m6A methylomic landscape in the lung tissues of ovalbumin-induced acute asthma mice, identified 127 hypermethylated and 43 hypomethylated differentially expressed mRNAs [11]. However, no further analyses of m6A regulatory factors have been performed. A study using a mouse model of allergic lung inflammation found that *IGF2BP2* predisposes macrophages to M2 polarization via an m6A dependency pattern, thereby attenuating allergic inflammation in the lung [12]. In another study, the expression of *METTL3*, T-bet, and *GATA3* was distinguished in bronchial epithelial cells from human and mouse asthma models, and *METTL3* increased Th2 differentiation and inhibited Th1 differentiation [13]. However, studies on m6A regulator-mediated RNA methylation modification and AR are scarce. Understanding the molecular mechanisms of respiratory allergies requires a combined analysis of both asthma and AR. The above-mentioned studies have not performed a panoramic analysis of m6A regulators of respiratory allergies, and there are no studies related to the nasal mucosa as the primary barrier of the respiratory tract against allergens.

Microarray analysis has been used to characterize m6A regulator expression, biological processes, and promising targets in childhood and severe asthma [14]. One study used predictive models and consensus clustering methods to identify m6A patterns and performed immune cell infiltration analysis for the two m6A patterns of asthma [15]. However, this study used only two predictive models to screen for key m6A regulators, which may overlook the impact of certain important factors on the disease while reducing the accuracy of prediction. Another study on severe asthma found 16 regulators differentially expressed, and two key m6A regulators (*YTHDF3* and *YTHDC1*). The results also revealed that *YTHDF3* and *EIF3B* affected the infiltration of eosinophils, which are vital in severe asthma [16]. However, the above two studies did not perform treatment analyses. Therefore, further comprehensive analyses are needed to identify the key m6A regulators that influence immune cell infiltration in respiratory allergies.

In this study, we aimed to screen key m6A regulators affecting immune microenvironment infiltration in respiratory allergies and the underlying mechanisms, as well as to perform drug sensitivity analysis of key m6A regulators, to reveal the molecular mechanisms of the unified airway theory of respiratory allergies and explore the mechanism of action of drugs.

## Materials and methods

### Data collection and construction of molecular subtypes in respiratory allergic diseases

The expression profiles of patients with respiratory allergic diseases were obtained from the published NCBI Gene Expression Omnibus database (GSE461712) (https://www.ncbi.nlm.nih.gov/geo/query/acc.cgi?acc=GSE46171) [17]. The GSE46171 dataset [18] contains 91 samples in common, including 11 from patients with AR, 17 normal samples, and 63 from patients with asthma; the species is *Homo sapiens*, the tissue source is nasal mucosa, and the gene sets of five RNA-modified genes (m6A, m1A, m5C, APA, and A-1) were obtained by Wang et al. [19], Chen et al. [20], and Cong et al. [21]. To analyze the differential expression characteristics of m6A regulators in patients with respiratory allergies, we used the "ConsensusClusterPlus" package (http://www.bioconductor.org/packages/release/bioc/html/ConsensusClusterPlus.html) [22] in R to cluster the samples according to the m6A regulators. The samples were divided into different groups by the expression of differentially expressed genes in each sample; the parameters were set to repeat 50 times (reps = 50), and the resampling rate was 80% (pItem = 0.8). The validity of the grouping was confirmed by principal component analysis (PCA) of the expression levels of all genes, and the results were visualized using the "ggplot2" package. Finally, we verified 22 m6A RNA methylation regulators in the molecular subtypes, including m6A writers, m6A readers, and m6A erasers: nine m6A writers (*METTL3*, *METTL14*, *WTAP*, *VIRMA*, *RBM15/15B*, *METTL16*, *CBLL1*, *ZC3H13*, and *ZCCHC4*), three m6A erasers (*ALKBH5*, *FTO* and *FMN*), and ten m6A readers (*YTHDF1*, *YTHDF2*, *YTHDF3*, *YTHDC1*, *YTHDC2*, *IGF2BPs*, *EIF3*, *HNRNPA2B1*, *HNRNPC*, and *HNRNPG*).

### Panoramic analysis of m6A regulators

To analyze the changes in m6A regulator expression in different clusters, we first extracted the expression of 22 m6A regulators in different clusters using custom Perl scripts. The "limma" package [23] in R was applied to identify and screen two sets of differentially expressed m6A regulators with significantly different clusters, and co-expression analysis of these m6A regulators was performed. We also identified the chromosomal localization of the m6A regulators, which was visualized with the "circos" package [24]. Finally, we observed the significantly different expression of the nine writers and three erasers in the two clusters in PCA.

### Construction of predictive models

Predictive models were constructed using two groups of differentially expressed m6A regulators with significantly different clustering subgroups. LASSO regression was performed on the training cohort using the R package "glmnet" [25]. The LASSO algorithm reduces the dimensionality of high-latitude data and constructs a model with fewer variables to explain the characteristics of the data [26]. A ten-fold cross-validation methodology was used to avoid overfitting the model built using the training cohort. Finally, a scoring system was constructed based on the regression coefficients computed using LASSO regression. Patients were assigned to high- or low-risk groups based on the cutoff value given by the R package "survival,” univariate Cox regression analysis was performed to screen the effective and significant m6A regulators, and the results were visualized using the R package "forestplot". Subsequently, a supervised machine learning algorithm was used to determine the differentially expressed m6A regulators in respiratory allergic diseases. To predict the impact of m6A regulators on the incidence of respiratory allergic diseases, we used the random forest (RF) algorithm to analyze the m6A regulators in the two clustering groups showing significant differences in PCA. RF is a machine learning algorithm for classification and regression that provides measures of variable importance, in contrast to single decision tree models, thereby making the results of our model more interpretable [27, 28]. Using the RF model, we constructed a multitude of decision trees, outputting the class label predicted by these trees, and the class prediction of the input factors was determined by majority vote. Each tree was built on a bootstrap training set that represented approximately two-thirds of the discovery cohort with replacements.

### Construction of molecular subtypes of hub m6A regulators

We used the hub m6A regulators to cluster the samples using the R package "ConsensusClusterPlus" [22] to divide the samples into different groups based on the expression of hub m6A regulators in each sample, with the following parameters: reps = 50 and pItem = 0.8. We also performed PCA, and the results were visualized using the R package "ggplot2". Finally, we validated the expression of the hub m6A regulators in the identified molecular subtypes.

### Construction of protein–protein interaction networks

A protein–protein (PPI) interaction network is composed of individual proteins that interact with each other [29]. Systematic analysis of the interactions of a large number of proteins in biological systems is important for understanding how proteins work in these systems: biological signaling, differential gene expression, energy and material cycle, and regulation in special physiological states such as diseases, and the functional connections between proteins. StarBase 2.0 (http://starbase-sysu.edu.cn/) [30] is a dataset that can systematically recognize RNA–RNA and protein–RNA interaction networks. We used this database to identify hub m6A regulator-protein regulatory relationships and visualized them using the Cytoscape software (version: 3.9.0).

### Functional enrichment analysis

Gene Ontology (GO) functional annotation analysis is a common method for large-scale functional enrichment studies of genes, including biological processes (BP), molecular functions (MF), and cellular components (CC). GO functional annotation analysis of the respiratory allergic disease-related hub m6A regulators was performed using the R software package “clusterProfiler” [31]. Gene set enrichment analysis (GSEA) was performed to assess the distribution trend of genes of a pre-ranked gene list in a gene table ranked by phenotype correlation, thereby evaluating their contribution to the phenotype [32]. We obtained the "c2.kegg.v7.4.symbols" and "c5.go.v7.4.symbols" gene sets in the MSigDB database (http://www.gsea-msigdb.org/gsea/index.jsp) [33] to perform GSEA and GO and Kyoto Encyclopedia of Genes and Genomes (KEGG) enrichment analyses using the "clusterprofiler" R package [31]. The KEGG is a widely used database for storing information on genomes, biological pathways, diseases, and drugs [34].

### Construction of subtypes with different immune characteristics

Single-sample GSEA (ssGSEA) was performed using the R package "GSVA", which is applied to assess the enrichment scores for each sample. We obtained 29 immune-related genes from Yin et al. [35], and assessed the level of immune cell infiltration in each sample based on the expression levels of immune cell-specific markers. We first determined the co-expression network for the 29 kinds of immune cells, and then cluster analysis was performed with the "ConsensusClusterPlus" package [22]. The samples were divided into different groups by evaluating their immune cell content. We also observed the expression of hub m6A regulators in different immune groups. The Pearson correlation coefficient between the expression levels of hub m6A regulators and number of immune cells was calculated, and the relationship between key m6A regulators and immune cell infiltration levels was evaluated. The screening conditions were correlation coefficients > 0.3 and *P* < 0.05.

### Identification of key m6A regulators and immune infiltration analysis

We performed immune cell infiltration analysis between nasal mucosal tissues of patients with respiratory allergic diseases using the CIBERSORT algorithm [36] to identify immune cells that were differentially enriched between different immune subgroups. CIBERSORT is an algorithm for deconvolution of the expression matrix of immune cell subtypes based on the principle of linear support vector regression, which was originally provided for the analysis of the tumor microenvironment and is now increasingly being used for the characterization of immune cell infiltration in non-tumor tissues [37]. It plays an important instructive role in making treatment decisions and predicting the prognosis of patients with respiratory allergic diseases. Key m6A regulators were screened using a support vector machine (SVM) algorithm. The support vector machine recursive feature elimination (SVM-RFE) machine learning algorithm [38] was used to analyze m6A regulators using two methods of grouping: one in which two groups were significantly differentiated from each other by PCA, and the other in which two groups corresponded to healthy individuals and patients with respiratory allergies. Key m6A regulators were obtained by identifying the overlapping genes in the LASSO and RF tree models with the characteristic genes in the above two groups. The SVM classifier was evaluated by calculating the area under the curve for the two groups. Finally, Pearson’s correlation coefficients between key gene expression levels and number of immune cells were calculated to evaluate the relationship between key genes and immune infiltration levels.

### Drug sensitivity analysis of hub m6A regulators

We obtained drug sensitivity data from the CellMiner database [39] for correlation analysis with differentially expressed m6A regulators. The CellMiner database integrates mutation, expression, and drug sensitivity data from the CCLE [40], COSMIC [41], and CellMiner consortia and provides dynamic drug sensitivity comparisons of external mutation or expression data, compared to other databases.

### Statistical analysis

All data calculations and statistical analyses were performed using R (https://www.r-project.org/, version 4.0.2). For the comparison of two groups of continuous variables, the significant differences between normally distributed variables were estimated by independent Student’s t-test, and the differences between non-normally distributed variables were analyzed using the Mann–Whitney U-test or Wilcoxon signed-rank test. All statistical P-values were two-sided, and *P* < 0.05 was considered statistically significant.

## Results

### Overall flow of experimental design

**Fig. 1:**
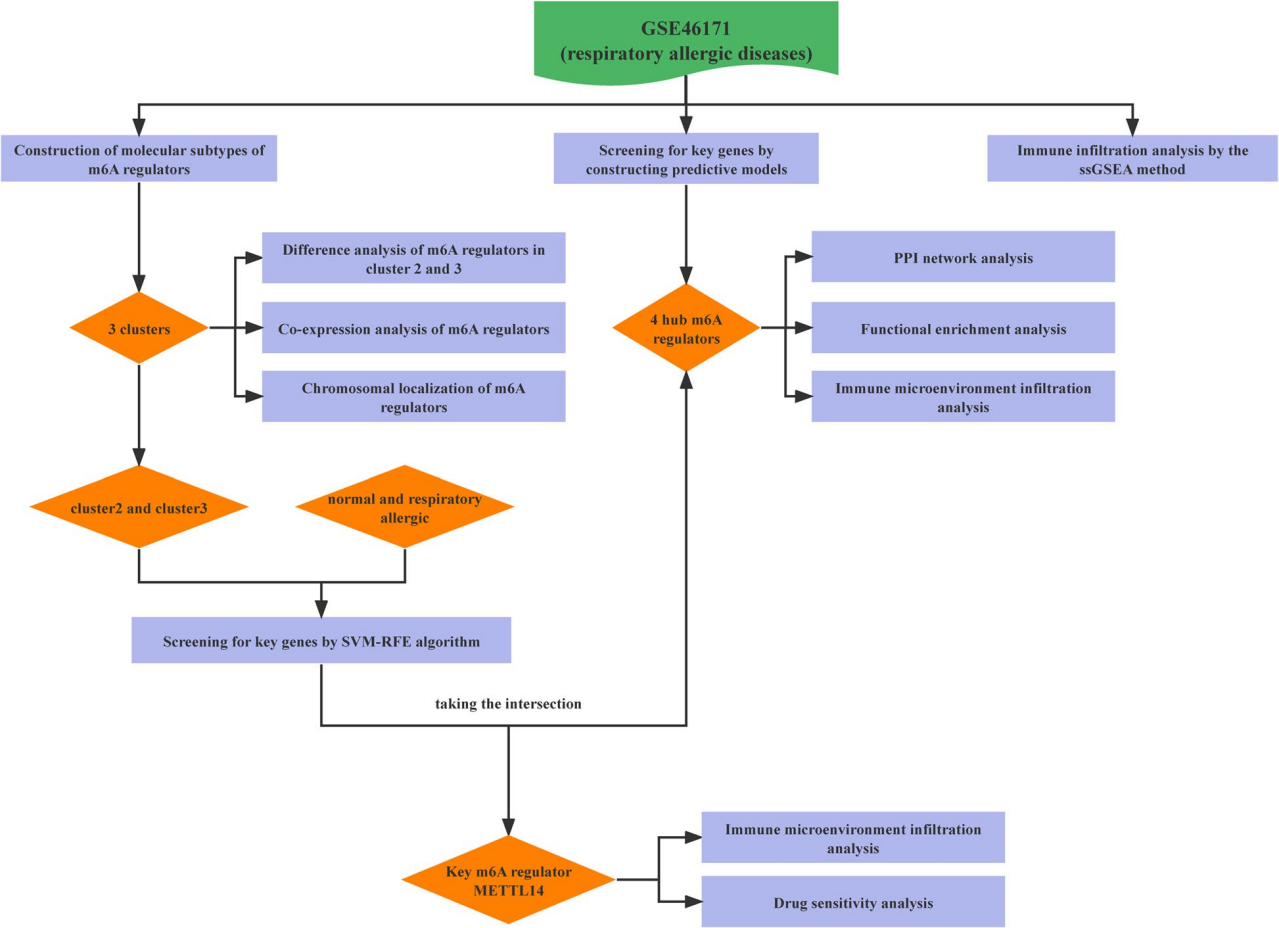
Flowchart of the study design

A flow diagram of this study is shown in Fig. 1. Briefly, we screened the expression levels of m6A regulators using the expression matrix of samples in the GSE46171 dataset and clustered all samples accordingly. Differential expression analysis was performed to observe the expression characteristics of m6A regulators between different clusters. LASSO and univariate Cox regression analyses were performed and RF model was used to screen hub m6A regulators between two clusters with significant differences in PCA, followed by further molecular typing and PPI and functional enrichment analyses for the four screened hub m6A regulators. Immune cell infiltration analysis was performed on the samples, which were grouped according to the immune infiltration characteristics to observe the expression characteristics of m6A regulators between different groups. In addition, we performed an immune microenvironment analysis to investigate the effects of hub m6A regulators on immune cell function in respiratory allergies. Finally, we screened the key m6A regulators by applying the feature selection SVM-RFE machine learning algorithm to identify the key gene METTL14, which was subjected to drug sensitivity analysis.

### Molecular typing of respiratory allergic diseases and overall expression characterization of m6A regulators

**Fig. 2:**
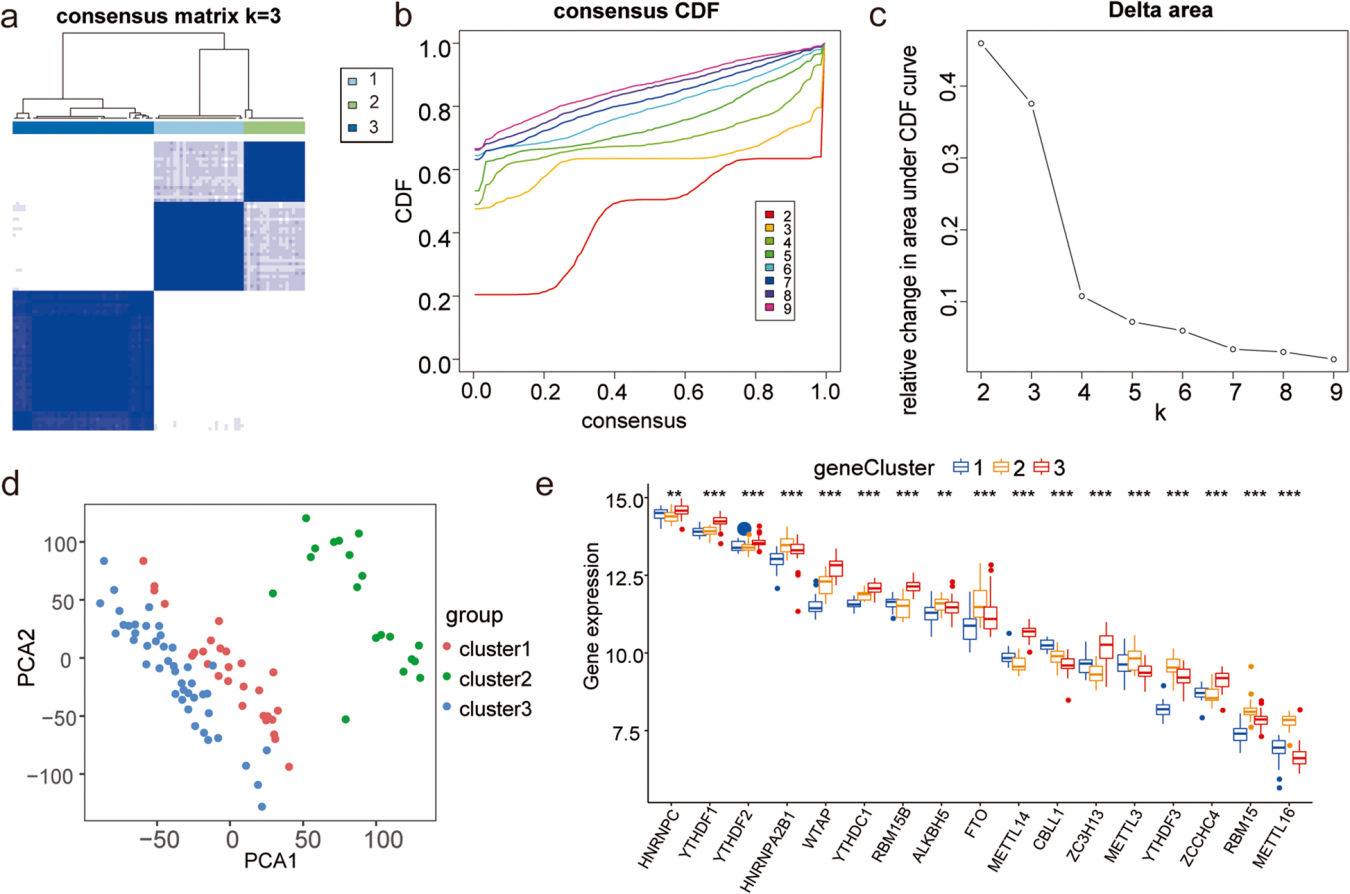
Molecular typing of respiratory allergic diseases. **a**-**c** Clustering of samples based on the expression of m6A regulators. **a** Consensus matrix showing cluster membership labeled by colored rectangles, enabling users to count the number of cluster members in the context of their consensus. **b** Cumulative distribution function (CDF) of the consensus matrices for each k (indicated by color). **c** Delta area plot showing the relative change of area under the CDF curve. **d** PCA of different clusters, where red is cluster 1, green is cluster 2, and blue is cluster 3. Clusters 2 and 3 had a high separation quality. (E) Differential expression of m6A regulators in different clusters; blue is cluster 1, yellow is cluster 2, and red is cluster 3. Asterisk indicates the statistical significance difference between cluster B and C. "**" means *p* < 0.01; "***" means *p* < 0.001

**Fig. 3:**
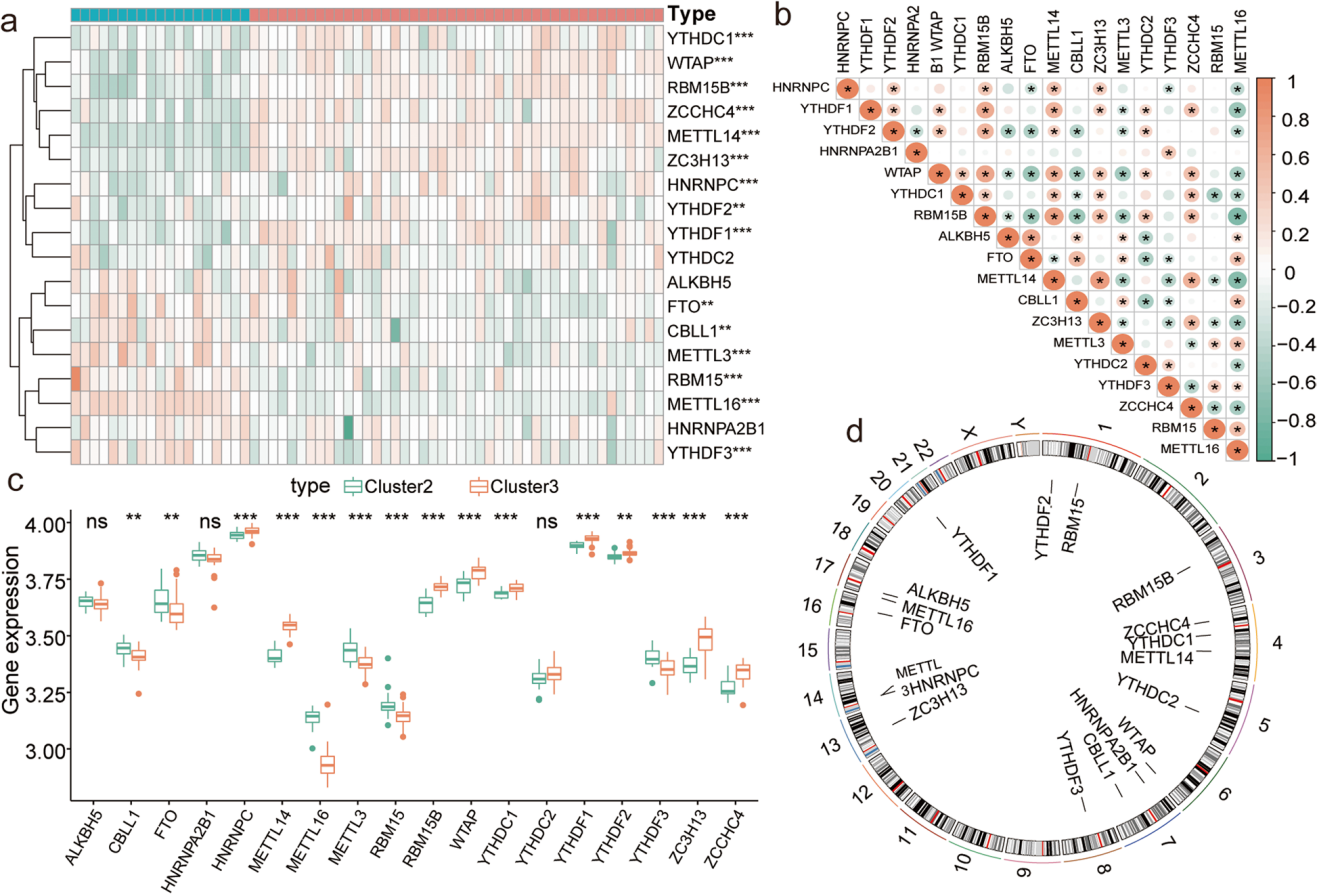
Gene expression and chromosomal localization of m6A regulators. **a** Heatmap of differential expression analysis of m6A regulators in clusters 2 and 3. **b** Co-expression analysis of m6A regulators; red indicates positive correlation, green indicates negative correlation, and circle size indicates correlation coefficient: the larger the circle, the higher the correlation, the smaller the circle, the smaller the correlation. asterisk indicates statistical significance of the correlation coefficient. "*" means *p* < 0.05. **c** Box plots of differential expression analysis of m6A regulators in clusters 2 and 3. Asterisk indicates the statistical significance difference between cluster 2 and 3. "**" means *p* < 0.01; "***" means *p* < 0.001. **d** Chromosomal localization of differentially expressed m6A regulators

**Fig. 4:**
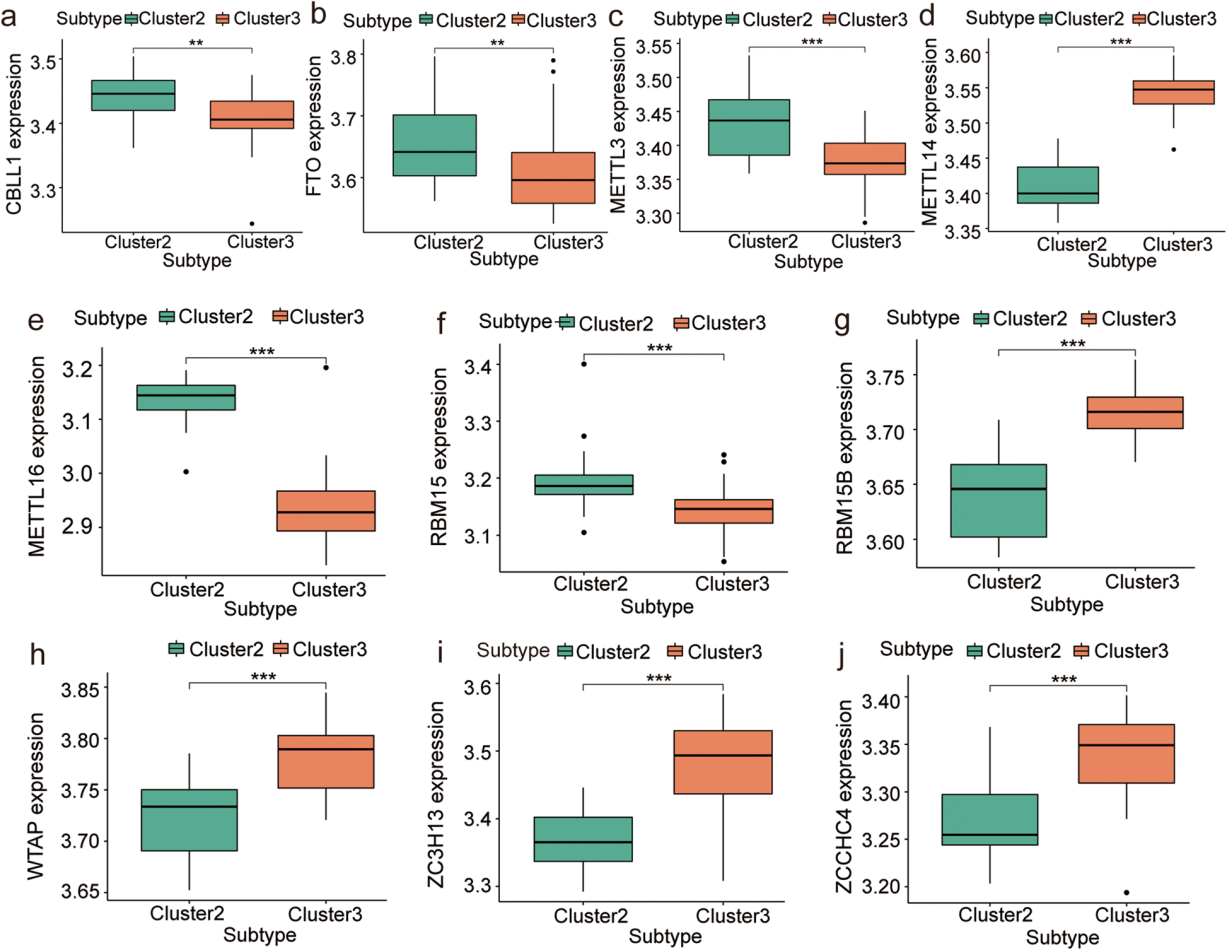
Differential expression analysis of m6A writers and erasers in clusters 2 and 3. (a-j) Differential expression analysis of CBLL1, FTO, METTL3, METTL14, METTL16, RBM15, RBM15B, WTAP, ZC3H13, and ZCCHC4 in clusters 2 and 3. Asterisk indicates the statistical significance difference between cluster 2 and 3. "**" means *p* < 0.01; "***" means *p* < 0.001

To explore the biology of m6A regulator expression in patients with respiratory allergies, we performed hierarchical clustering of all samples based on the expression levels of m6A regulators. All samples were divided into three groups (cluster 1: *n* = 28; cluster 2: *n* = 19; cluster 3: *n* = 44, Fig. 2a and c). Cumulative distribution function (CDF) of the consensus matrices for each k (Fig. 2b). Differential expression analysis led to the identification of *HNRNPC*, *YTHDF1*, *YTHDF*2, *HNRNPA2B1*, *WTAP*, *YTHDC1*, *RBM15B*, *ALKBH5*, *FTO*, *CBLL1*, *ZC3H13*, *METTL3*, *YTHDF3*, *ZCCHC4*, *RBM15*, *METTL16*, and *METTL14* as m6A regulators (*P* < 0.01; Fig. 2e). PCA showed that clusters 2 and 3 had a high separation quality (Fig. 2d). Therefore, we performed differential analysis of clusters 2 and 3, and the results show that the expression levels of *HNRNPC*, *YTHDF1*, *YTHDF2*, *WTAP*, *YTHDC1*, *RBM15B*, *FTO*, *CBLL1*, *ZC3H13*, *METTL3*, *YTHDF3*, *ZCCHC4*, *RBM15*, *METTL16*, and *METTL14* were significantly different between these two clusters (Figs. 3a, c, and 4). Co-expression analysis showed high expression correlations between many m6A regulators, and the vast majority of m6A regulators such as *METTL14*, *HNRNPC*, *YTHDF1*, *YTHDF2*, *WTAP*, and *YTHDC1* had significant correlations (Fig. 3b). The results of chromosome localization analysis are shown. (Fig. 3d). *RBM15* is located on chromosome 1, *RBM15B* on chromosome 3, *METTL14* on chromosome 4, and *METTL3* on chromosome 14.

### Construction of respiratory allergic disease predictive models

**Fig. 5:**
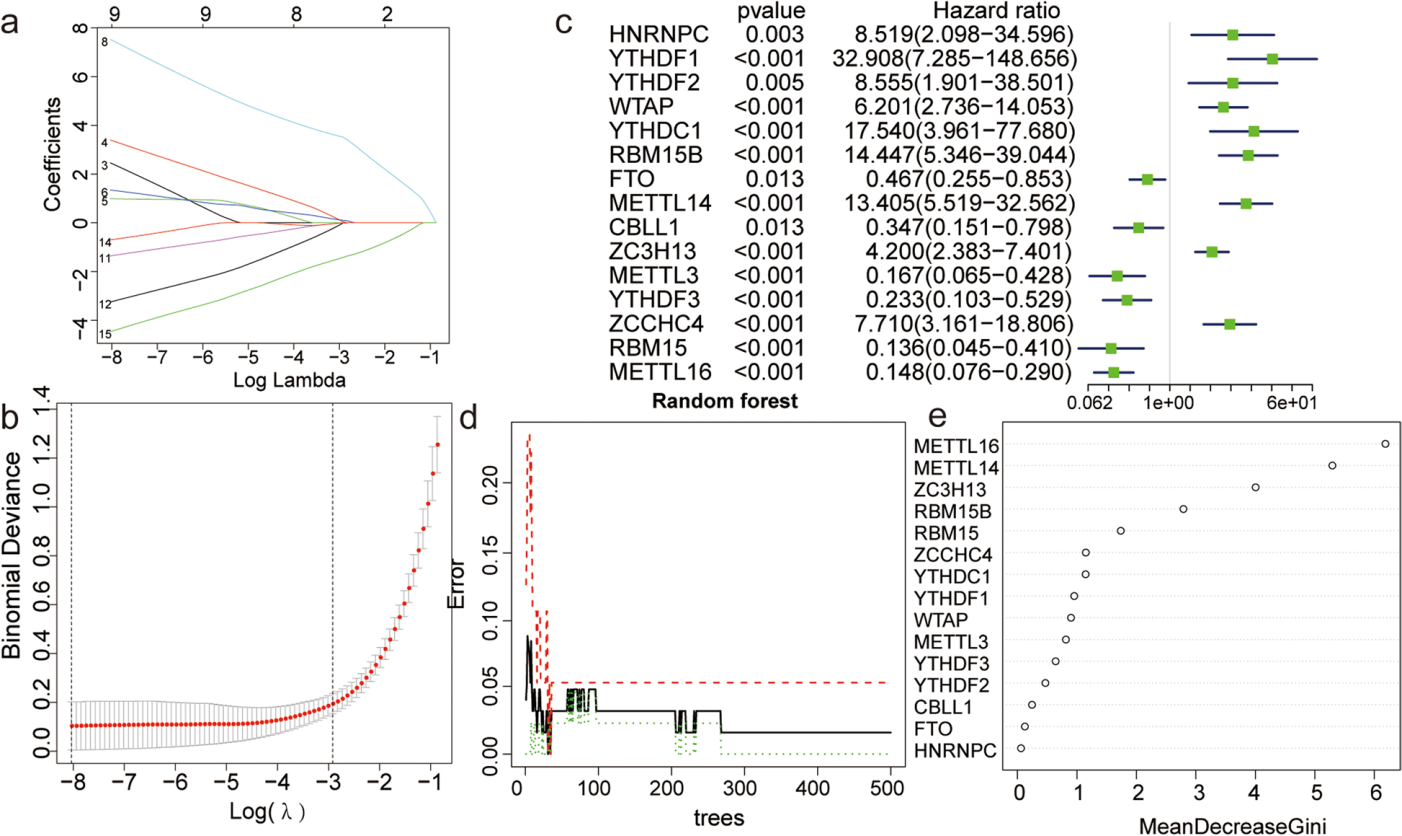
LASSO Cox regression analysis and random forest model for hub m6A regulator screening. **a**-**b** Construction of LASSO predictive model based on m6A regulators. **c** Forest plot for Cox proportional hazards regression model. **d**-**e** Construction of random forest model based on five m6A regulators

We used the LASSO regression algorithm and RF algorithm and performed Cox regression analysis to construct AR predictive models based on the expression levels of m6A regulators (Fig. 5a-e). We constructed LASSO predictive models containing nine m6A regulators, *YTHDF2*, *WTAP*, *YTHDC1*, *RBM15B*, *METTL14*, *METTL3*, *YTHDF3*, *RBM15*, and *METTL16* (Fig. 5a-b), and an RF model with five m6A regulators, *METTL14*, *METTL16*, *RBM15*, *RBM15B*, and *ZC3H13* (Fig. 5d-e). Finally, the genes present in both models were the hub m6A regulators *METTL14*, *METTL16*, *RBM15B*, and *RBM15*.

### Molecular typing of respiratory allergic diseases and overall expression characterization of hub m6A regulators

**Fig. 6:**
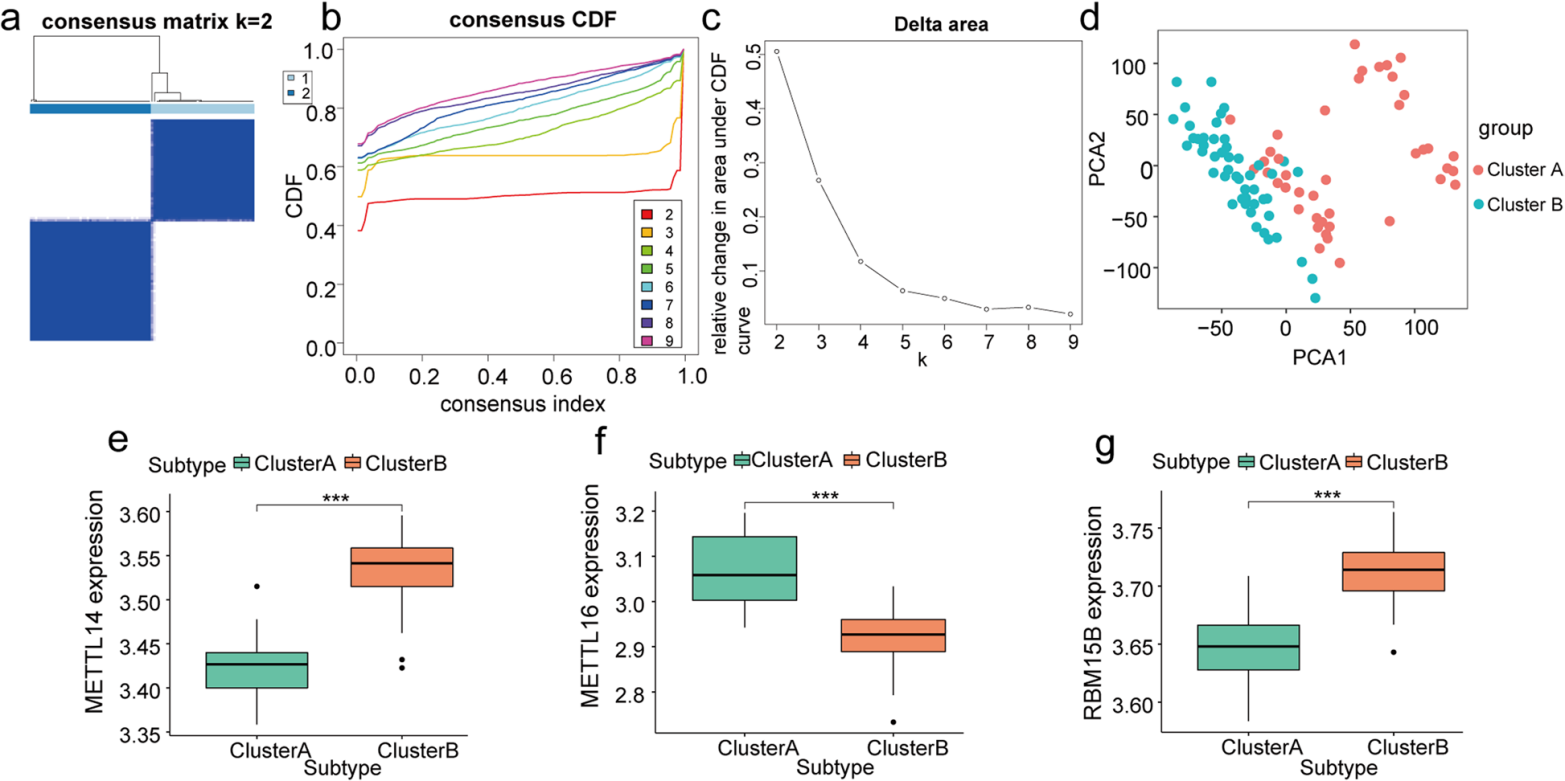
Molecular typing of respiratory allergic diseases by hub m6A regulators. **a** Consensus matrix showing cluster membership marked by colored rectangles, enabling users to compute the number of cluster members in the context of their consensus. **b** Consensus cumulative distribution function (CDF) of the consensus matrix for each k (indicated by color). **c** Delta area plot showing the relative change of area under the CDF curve; (**d**) PCA under different groupings, where cluster A is in red, and cluster B is in blue. **e**–**g** Differential expression of METTL14, METTL16, and RBM15B under different clusters, where cluster A is in green, and cluster B is in red. Asterisk indicates the statistical significance difference between cluster A and B. "***" means *p* < 0.001

We performed hierarchical clustering of all samples based on the expression of the four hub m6A regulators, and all samples were divided into two different subtypes (cluster A: *n* = 42; cluster B: *n* = 49; Fig. 6a-c). PCA results showed a high separation quality between the two clusters (Fig. 6d). The results of the difference analysis between the two clusters show that *METTL14*, *METTL16*, and *RBM15B* were significantly differentially expressed between the two clusters (*P* < 0.001; Fig. 6e-g).

### Construction of a PPI network of m6A regulators

We constructed a PPI network of hub m6A regulators, which contained four hub m6A regulators and 456 interacting proteins (Table S1). Among them, *RBM15B* had the most interacting proteins (278), followed by *METTL16* (106), *RBM15* (57), and *METTL14* (32).

### Functional enrichment analysis of m6A regulators

We performed functional enrichment analysis on the four hub m6A regulators, including GO (Table S2) analysis and GSEA (Table S3). As shown in Table S2, hub genes were mainly enriched in biological processes such as macromolecule methylation, nuclear envelope, nuclear speckle, catalytic activity, and transferase activity. As shown in Table S3, *METTL14* was enriched in functions such as glutathione derivative metabolic process and glutathione metabolic process; *METTL16* was enriched in negative regulation of epithelial cell differentiation and respiratory chain complex; *RBM15* was enriched in adherens junction and calcium signaling pathway; and *RBM15B* was potentially associated with glutathione derivative metabolic process, positive regulation of calcium ion transmembrane transporter activity, and other functions.

### Analysis of immune cell infiltration in respiratory allergic diseases

**Fig. 7:**
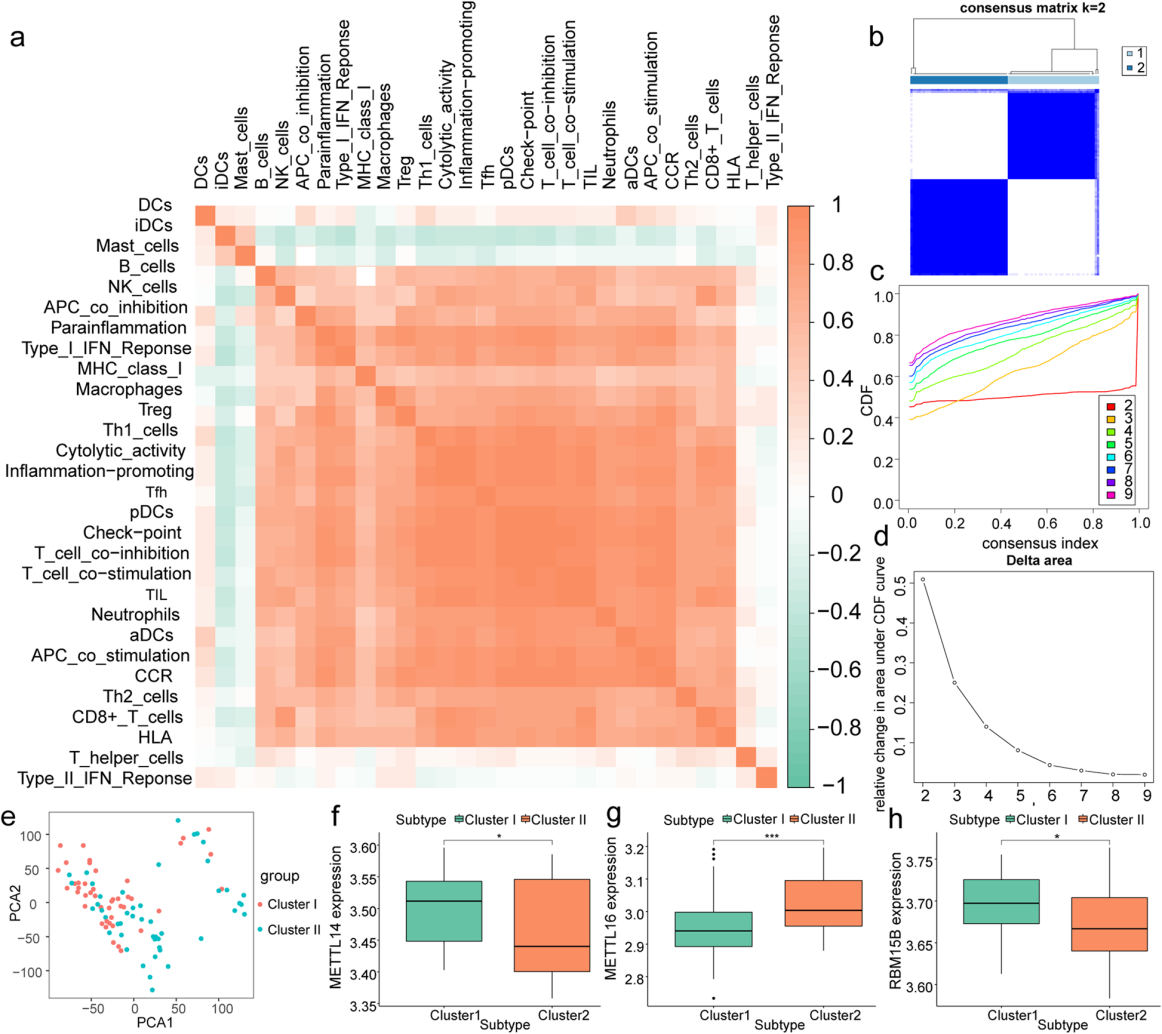
Immune cell infiltration analysis of respiratory allergic diseases. **a** Co-expression analysis of the degree of infiltration of 29 immune cell types in nasal mucosa tissues, with positive correlation in red and negative correlation in green; b-d: clustering grouping based on 29 immune cells. **b** Consensus matrix showing cluster membership labeled by colored rectangles, enabling users to count the number of cluster members in the context of their consensus. **c** Cumulative distribution function (CDF) of the consensus matrices for each k (indicated by color). **d** Delta area plot showing the relative change of area under the CDF curve. **e** PCA under different groupings, where cluster I is in red, and cluster II is in blue. **f**–**h** Differential analysis of hub m6A regulators between different immune clustering groups. Green for cluster I, red for cluster II. Asterisk indicates the statistical significance difference between cluster I and II. "*" means *p* < 0.05; "***" means *p* < 0.001

To analyze the immune cell infiltration characteristics in the nasal mucosal tissues of patients with respiratory allergies, we first calculated the degree of infiltration of 29 immune cell types in each tissue using the ssGSEA method. Co-expression analysis showed that the number sof Th1, Tfh, and other cells were highly correlated with those of most immune cells (Fig. 7a). Next, we performed hierarchical clustering of all samples based on the expression of 29 immune cell gene sets into two different subtypes (I: *n* = 44; II: *n* = 47; Fig. 7b-d), and the results of PCA showed a high quality of separation (Fig. 7e). Differential analysis showed significant differences in the expression of *METTL14*, *METTL16*, and *RBM15B* between groups I and II (*P* < 0.05) (Fig. 7f-h).

**Fig. 8:**
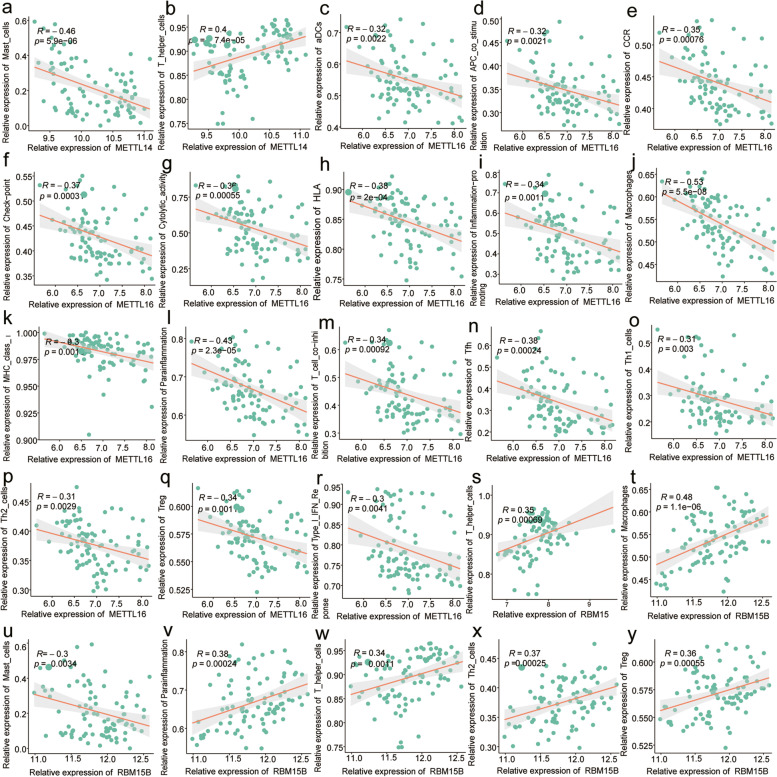
Correlation analysis of hub m6A regulators and immune cells. The slope is the degree of the correlation, and the p-value indicates the significance level. Pearson correlation coefficients (R) and *p* values are presented

Next, we performed immune microenvironment analysis to investigate the function of hub m6A regulators in respiratory allergies. By correlating the expression of individual immune indices calculated using ssGSEA with hub m6A regulators, we found that most hub genes were associated with the number of macrophages, and mast cells, Th2 cells and that various other innate and acquired immune cells were significantly negatively correlated with the degree of infiltration, with macrophages showing the highest negative correlation with METTL16 expression (-0.53, Fig. 8).

**Fig. 9:**
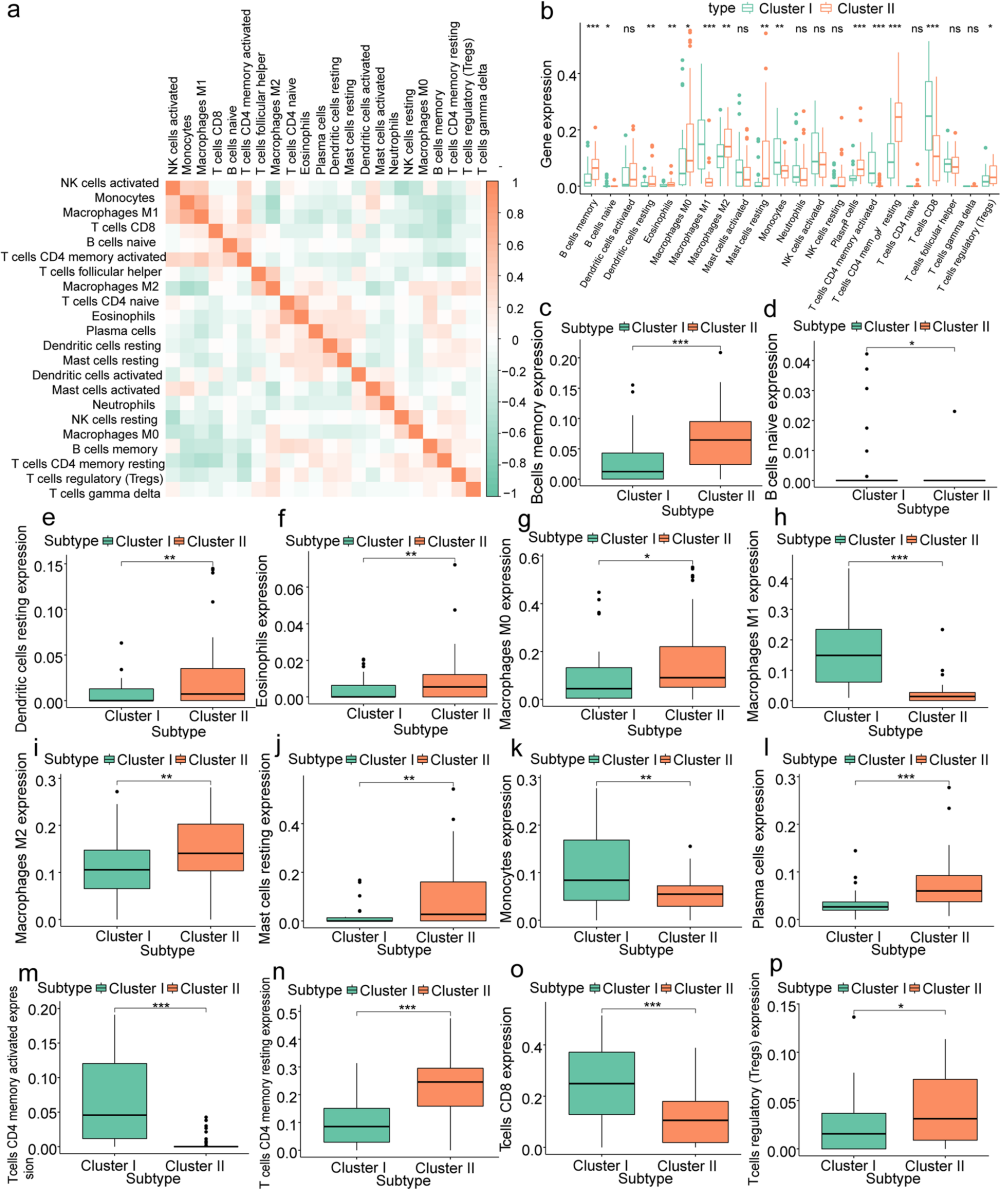
CIBERSORT calculation of the number of immune cells in each sample. **a** Co-expression analysis of 22 immune cell types, red stands for positive correlation, green stands for negative correlation. **b**-**p** Expression of 22 immune cell types in different immune characteristic subtypes; cluster I stands for green, cluster II stands for red. Asterisk indicates the statistical significance difference between cluster I and II. "*" means *p* < 0.05; "**" means *p* < 0.01; "***" means *p* < 0.001

Finally, we used the CIBERSORT algorithm to validate the results of the degree of tissue immune cell infiltration calculated using the ssGSEA method, which showed that monocytes and M1 macrophages were highly correlated with most immune cells (Fig. 9a). Differential analysis showed that 14 of 22 immune cells, memory B cells, naïve B cells, dendritic cells, eosinophils, M0 macrophages, M2 macrophages, resting mast cells, plasma cells, CD4 + memory activated T cells, and Tregs were more infiltrated in cluster 2, whereas CD8 + T cells, CD4 + memory resting T cells, monocytes, and M1 macrophages were more infiltrated in cluster 1 (*P* < 0.05, Fig. 9b-p). These findings suggest the heterogeneity of respiratory allergic diseases from the perspective of immune cell infiltration.

### Key m6A regulator screening and immune cell infiltration analysis

**Fig. 10:**
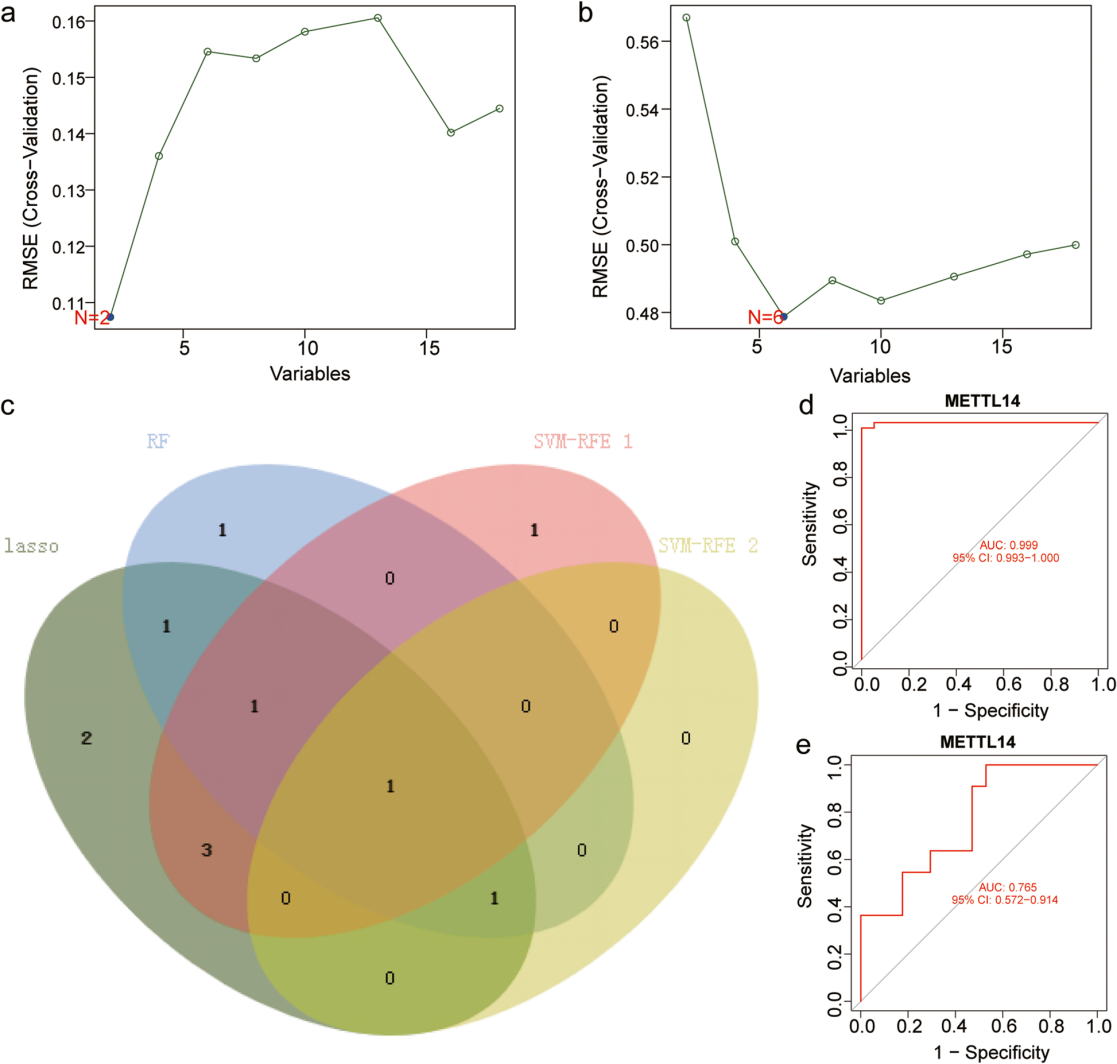
Screening of key m6A regulators. **a**-**b** Key m6A regulators were analyzed by applying the feature selection support vector machine recursive feature elimination (SVM-RFE) machine learning algorithm in clusters 2 and 3 and healthy individuals and patients with respiratory allergies to screen the signature genes in two comparisons. **a** Clusters 2 and 3. **b** Healthy individuals and patients with respiratory allergies. **c** Venn diagram was plotted for the intersection of nine m6A regulators in the LASSO regression model (green), four m6A regulators in the RF analysis (blue), two signature genes in the clusters 2 and 3 (pink), and six signature genes in the healthy individuals and patients with respiratory allergies (yellow). **d**-**e** ROC curves for METTL14 were plotted in the clusters 2 and 3 and healthy individuals and patients with respiratory allergies. The area under the ROC curves and the associated 95% CIs are shown

To further screen key genes among hub m6A-regulators, we used the SVM-RFE machine learning algorithm to analyze m6A regulators in clusters 2 and 3 and healthy individuals and patients with respiratory allergies and screened the signature genes in both comparisons (Fig. 10a and b), where *METTL14* and *METTL16* were the signature genes for clusters 2 and 3, and *METTL3*, *YTHDF1*, *METTL14*, *YTHDC1*, *RBM15*, and *YTHDF3* were the signature genes for the healthy individuals and patients with respiratory allergies. Subsequently, we obtained the key gene *METTL14* by taking the intersection of the nine m6A regulators in the LASSO model, the four m6A regulators in the RF tree model, and the characteristic genes in the above two comparisons (Fig. 10c). Finally, we plotted the receiver operating characteristic (ROC) curves of the key gene *METTL14* in the two comparisons (Fig. 10d-e).

**Fig. 11:**
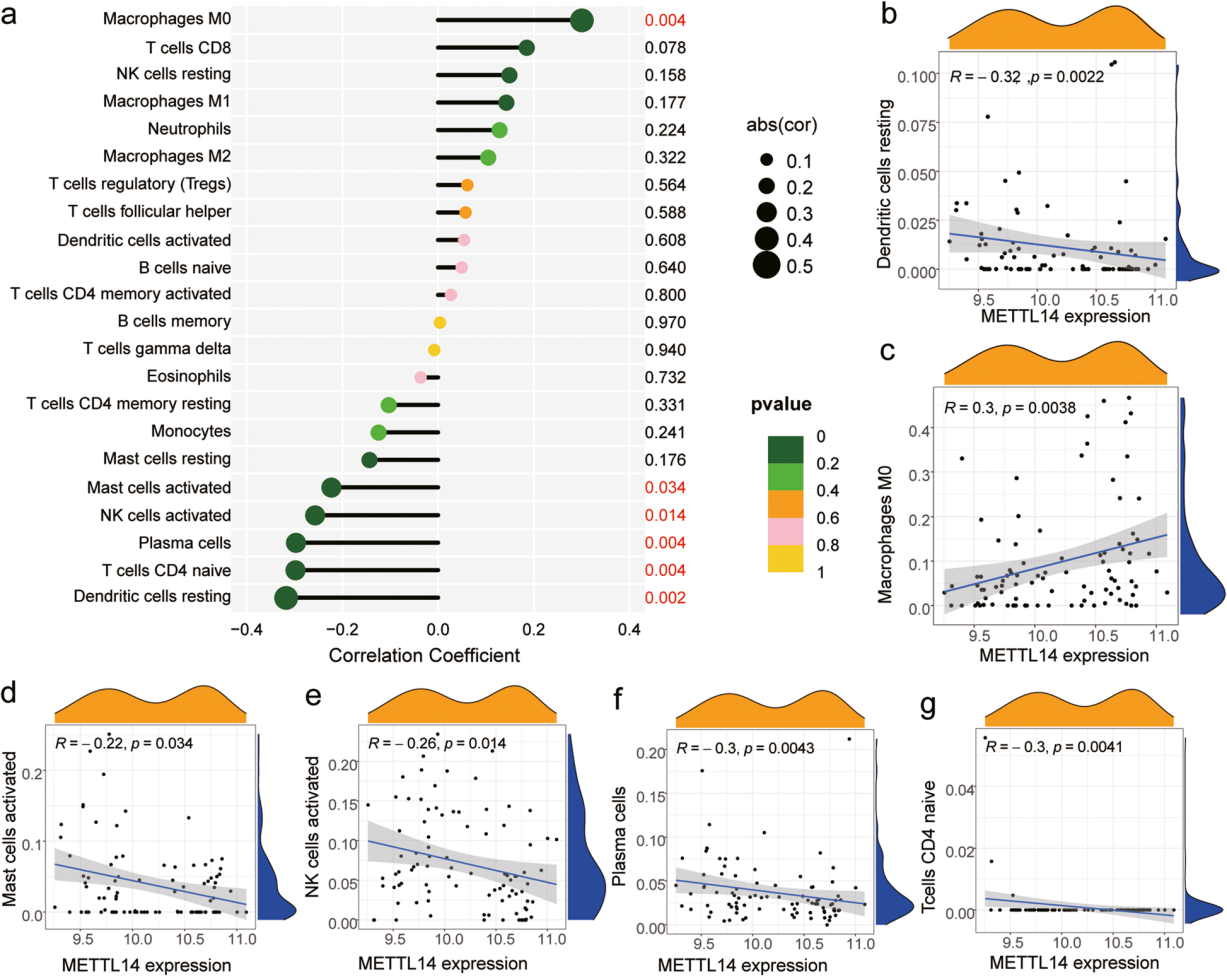
Immune cell infiltration analysis of METTL14. **a** Overall correlation analysis of 22 immune cell types with METTL14 expression; different circle colors represent different *p*-values, and circle size indicates correlation coefficient. **b**-**g** Correlation analysis of METTL14 expression and immune cells: M0 macrophages, activated mast cells, activated NK cells, plasma cells, naïve CD4 + T cells. Pearson correlation coefficients (R) and p values are presented

Correlation analysis of the key gene *METTL14* with 22 immune cells showed that six of them were correlated with *METTL14* expression (Fig. 11a). Among them, the degree of infiltration of M0 macrophages was positively correlated with *METTL14* expression, whereas that of dendritic cells, mast cells, NK cells, and CD4 + T cells had a negative correlation with *METTL14* expression (Fig. 11B-G).

### Drug sensitivity analysis of METTL14 expression

**Fig. 12:**
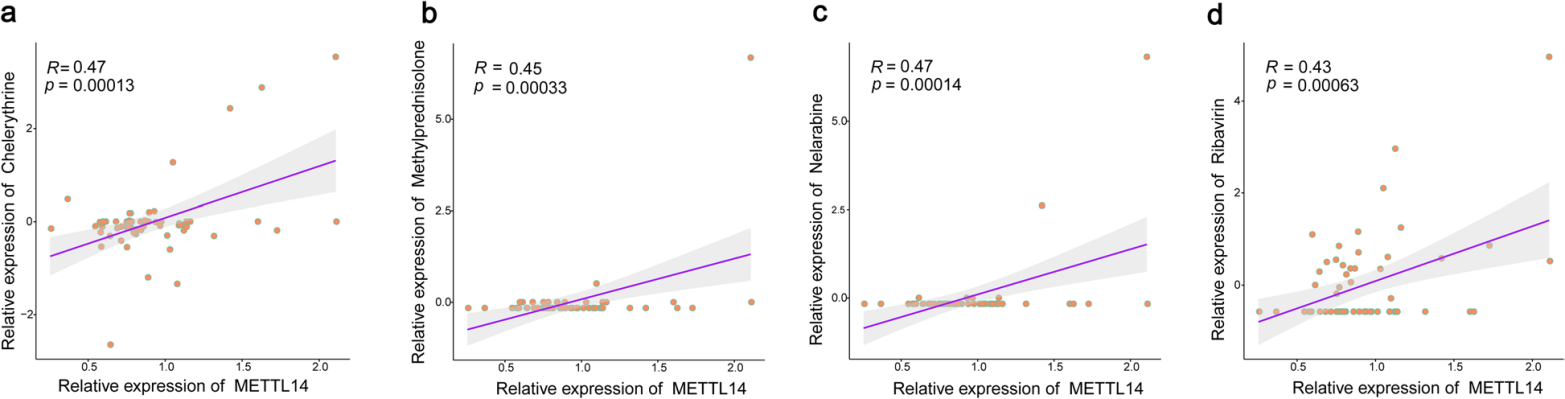
Drug sensitivity analysis of key m6A regulator METTL14. Dots indicate the samples, the slope represents the magnitude of the correlation, and the *p*-value indicates the significance level. Pearson correlation coefficients (R) and p values are presented

Drug sensitivity analysis revealed significant correlations between *METTL14* expression and chelerythrine, methylprednisolone, nelarabine, and ribavirin(Fig. 12). The correlation coefficient of *METTL14* with methylprednisolone was 0.45 (Fig. 12b).

## Discussion

AR and asthma have been previously evaluated and treated as separate diseases; however, current clinical studies have shown that both the presence and severity of rhinitis are associated with worse asthma prognosis [42]. Consequently, the unified airway theory has gained significant attention in recent times, but there is a paucity of research on the molecular mechanisms that underlie its pathophysiology. The current treatment regime focused only on alleviating the symptoms during exacerbations, and there in no reliable treatment available. Hence, comprehending the pathology and molecular mechanisms of respiratory allergies can aid in clinical diagnosis and treatment. While previous studies have focused on m6A regulation of severe asthma [16] and its biological function [43], but little attention has been given to this common airway problem. First of all, our study focused on patients with both allergic rhinitis and asthma. Secondly, we analysed the molecular classification of respiratory allergic diseases and conducted corresponding bioinformatics analyses. This allowed us to better understand the direction of treatment and guide the selection of drugs. Third, we opted to use nasal mucosa samples instead of whole blood samples. Nasal mucosa serves as a physical barrier in the respiratory tract and is crucial in the early detection and treatment of diseases. In this study, to explore the regulatory mechanisms of m6A regulators in the immune microenvironment of respiratory allergic diseases, we performed a comprehensive analysis of m6A regulators in nasal mucosa samples from 11 patients with AR, 17 normal samples, and 63 patients with asthma, to obtain hub m6A regulators. Functional enrichment and immune microenvironment infiltration analyses were then performed on hub m6A regulators, followed by the drug sensitivity analysis on the hub m6A regulator *METTL14* was performed.

We identified three patterns of m6A RNA methylation mediated by 22 m6A regulators. Next, the screening range of hub m6A regulators was narrowed down, resulting in three hub genes affecting respiratory allergies: the three "writers" *METTL14*, *METTL16*, and *RBM15B*. *METTL14* mainly has a SAM-binding domain and an EPPL motif required for m6A methylation, as well as a coiled-coil domain for mediating protein–protein interactions and a G-rich sequence at the N- and C-terminal ends [44]. *METTL14* acts as an allosteric activator and enhances the catalytic activity of *METTL3* [45]. In addition, *METTL14* is localized on chromosome 4, and the TLR10-TLR1-TLR6 locus, located in a 90 kbp region on chromosome 4, has been reported to be associated with the pathogenesis of AR [46]. Some specific regions on chromosome 4 are also likely to contain susceptibility genes for AR [47]and asthma [48]. *METTL16* harbors two conserved functional domains, the Rossmann-like fold of class I methyltransferases and a SAM-binding domain as the methyl donor [49]. It has a high structural similarity to the *METTL3*/*14* complex, but as a novel player, it also contains additional regions; the role that *METTL16* plays in its life cycle has not been determined [50]. *METTL16* is localized on chromosome 17, and genetic alterations of the 17q12-21 locus are associated with allergic asthma, according to previous findings [51]. Both *RBM15* and *RBM15B* consist of three RNA recognition motif (RRM) domains. *RBM15* and its paralog *RBM15B*, which serve as adapter proteins, bind and recruit the *WTAP*-*METTL3* complex to U-rich regions [52]. *RBM15B* is located on chromosome 3, and the 3q13, 3q1313.31, and 3p24 loci have been identified to be correlated with AR [53–55]. The above study validates our findings in one respect, indicating that hub m6A regulators have a relationship with respiratory allergic diseases.

To further elucidate the downstream molecular mechanisms and functions of respiratory allergy-related m6A regulators, we conducted a functional enrichment analysis on four hub genes. Our GO analysis demonstrated that they these genes were primarily associated with methyltransferases. Furthermore, GSEA results revealed that over three pathways were linked to the progression of respiratory allergies, including glutathione derivative metabolic processes, calcium signalling pathways, and regulation of autophagy. For example, Beier et al. [56] found that total glutathione levels are considerably higher in patients with mild and moderate asthma than in healthy individuals. Previous studies have shown that disturbances in oxidation/reduction (redox) reactions and impaired antioxidant defenses are risk factors for the development and severity of asthma [57]. The critical antioxidant defense of the airway epithelial surface against reactive oxygen and nitrogen species is provided by extracellular glutathione peroxidase (eGPx), and glutathione is an essential cofactor for eGPx activity [58]. The effect of glutathione on the regulation of Treg/Th17 cell homeostasis through inhibition of intracellular autophagy has also been demonstrated in peripheral blood mononuclear cells (PBMCs) from patients and mouse models of AR [59]. This is consistent with our data mining results from lncRNA microarray datasets GSE65204 and GSE19187, which also identified an effect of glutathione on respiratory allergy [60]. Mast cell degranulation plays a key role in allergic responses to conditions such as asthma and AR [61]. This increase in cytosolic Ca^2+^ influx probably activates mast cell degranulation [62]. A fundamental aspect of asthma pathophysiology is the elevation of intracellular calcium ion concentrations, and calcium-sensitive receptor antagonists may alleviate airway hyper-responsiveness and inflammatory responses in patients with asthma [63]. Microarray analysis of DNA methylation profiles [64] in patients with asthma and lncRNA expression profiles [65] in mice with AR also identified the calcium signaling pathway as a key pathway affecting respiratory allergies. Previous studies have verified the effect of autophagy on asthma, both in the lung tissue of a mouse model of severe asthma [66] and in the macrophages of patients with asthma [67]. Some autophagy-related genes are also involved in the pathogenesis of asthma [68].

Furthermore, PPI results of the four hub m6A regulators showed that a considerable number of the interaction genes were involved in the calcium signaling pathway, glutathione metabolism, and autophagy. Some calcium signaling pathway-related genes, such as *ERBB2, PDE1B, PDGFA, TPCN1,* and *MCU*, have been reported to participate in the development of allergic diseases [69–73]. Recent studies have also verified that PGD regulates glutathione metabolism and SQSTM1 affects autophagy in allergic diseases [74, 75]. However, whether m6A modifications mediated by m6A regulators affect the expression of genes related to the above pathways and thus promote respiratory allergies remains to be verified.

Studies have demonstrated that respiratory allergic diseases are complex chronic airway inflammatory diseases characterized by the infiltration of multiple inflammatory cells; therefore, we analyzed immune cell infiltration in all samples. The results showed significant differences in the expression of *METTL14*, *METTL16*, and *RBM15B* in the different groups based on the expression of 29 immune cell gene sets. Next, we performed a correlation analysis between the expression of m6A regulators and the index of infiltrating immune cells, which suggested that many immune cells are co-regulated by m6A regulators. For example, mast cells are closely associated with respiratory allergies; m6A regulators bind to the high-affinity receptor FcεR1 on mast cells and trigger an immediate hypersensitivity response that is critical in the pathogenesis of AR and allergic asthma [76]. Our findings show that the degree of mast cell infiltration was negatively correlated with *RBM15B* and *METTL14* expression. Another example is Th2 cells, whose hyperactivation is key to respiratory allergy [77], whereas the number of Th2 cells was negatively correlated with *METTL16* expression and positively correlated with *RBM15B* expression in our results. Therefore, we hypothesized that the expression of *METTL14*, *METTL16*, and *RBM15B* closely influences the pathogenesis of respiratory allergies in mast and Th2 cells. In addition, the strongest negative correlation between the number of macrophages and *METTL16* expression was observed in our study. To our knowledge, this is the first report of a significant correlation between *METTL16* expression and the number of macrophages in respiratory allergic diseases. Previous studies have demonstrated that M0 macrophage polarization is regulated by m6A modification, and *METTL3*, by upregulating STAT1 expression through methylation of STAT1 mRNA, strongly promotes the polarization of M0 macrophages to M1 macrophages and, conversely, inhibits the polarization of M2 macrophages [78]. M2 macrophages are important for relieving symptoms of asthma, and excessive M2 macrophages may promote inflammatory responses, cell proliferation, and mucus secretion, and cause airway spasms [79]. Furthermore, *METTL16* is involved in the regulation of *METTL3*/*METTL14*-induced activities [80]. Therefore, the above experimental study established a similar correlation between *METTL16* expression and the number of macrophages. However, the molecular mechanism by which *METTL16* regulates macrophages in respiratory allergies requires further in-depth study. Notably, our work differs from previous studies in that a combined analysis of asthma and AR established a new relationship between m6A and immune cell infiltration in respiratory allergies, expanding our understanding of the pathogenesis of these diseases.

Finally, we applied various algorithms to analyze the intersection of the foregoing hub genes to identify the key gene *METTL14*, suggesting that *METTL14* is a key m6A regulator for respiratory allergies by plotting the ROC curve of *METTL14*. In this study, we used multiple approaches for predictive modeling and SVM algorithms to cluster the samples from multiple perspectives, thus narrowing down the screening range of key genes and avoiding the lack of accuracy associated with single-dimensional screening methods. Immune cell infiltration analysis of *METTL14* revealed that the degree of M0 macrophage infiltration was positively correlated with *METTL14* expression, whereas the number of CD4^+^ T cells was negatively correlated with *METTL14* expression. It has been shown that the deletion of *METTL14* in CD4^+^ T cells promotes the destabilization of Socs1, Socs3, and Cish mRNAs, preventing their homeostatic proliferation and differentiation to Th1 and Th2 cells [81]. Dysregulation of the Th1/Th2 balance is an important factor in the development of allergic reactions [82]. In addition, *METTL14* recognizes *IGF2BP2* (m6A reader), and the binding of *IGF2BP2* to PPARγ enhances the stability and translation of PPARγ mRNA, thereby enhancing IL-4-induced activation of M2 macrophages [12]. The above findings are consistent with our data mining results; therefore, our study conclusively identified *METTL14* as a key m6A regulator involved in the regulation of the immune microenvironment in respiratory allergic diseases.

In drug sensitivity analysis, we found that methylprednisolone was closely associated with *METTL14* expression. The other three drugs, chelerythrine, nelarabine, and ribavirin have potential anti-inflammatory and antioxidant effects and may impact the immune system, their toxicity has prevented their clinical use in treating respiratory allergic diseases. Methylprednisolone is a glucocorticoid steroid, and numerous studies have demonstrated its utility in relieving respiratory allergy symptoms through both oral and topical routes of administration [83]. The type of sample selected for this study was nasal mucosal tissue; therefore, we speculated whether topical nasal glucocorticoids could improve symptoms associated with both the upper and lower airways in respiratory allergies. However, our hypothesis has indeed been tested in clinical applications, as nasal glucocorticosteroids delivered into the nasal cavity can reverse this increased airway hyperresponsiveness in allergy-related lower airways in patients with AR and asthma and decrease the dose of inhaled corticosteroids [84]. Although some studies have suggested that the mechanism underlying this phenomenon is neural modulation [85], it is difficult to explain all the mechanisms underlying the relation between upper respiratory tract inflammation and asthma using one theory. Our study provides new insights into this phenomenon. Therefore, further studies are needed to investigate whether topical nasal methylprednisolone improves respiratory allergic symptoms by modulating *METTL14* expression and its underlying mechanism.

However, a few limitations should be acknowledged. First, the lack of detailed clinical outcomes of the study subjects makes it impossible to combine clinical information for analysis. Second, the sample size was small, and patients with both AR and asthma were not included. Third, our bioinformatic results require validation through further wet-lab testing. We are planning a corresponding experiment. We will perform western blotting, real-time quantitative PCR, and immunohistochemistry assays to identify the expression of hub m6A regulators to validate our bioinformatic predictions. Next, to elucidate the role of m6A regulators in respiratory allergies, differential phenotypic and methylation analyses should be performed by knocking out or overexpressing genes encoding methylation-related enzymes, and gene-level analysis should be performed by combining MeRIP sequencing to identify potential target genes regulated by m6A. Currently, we have identified experimental verifications of the function of immune cells that relate to the key m6A regulatory factor METTL14 in other diseases [86–88]. This discovery supports our research efforts.

## Conclusions

We investigated the gene expression features and potential functions of m6A regulators in respiratory allergic diseases by using a series of bioinformatics techniques for the first time and identified *METTL14* as a key m6A regulator affecting the immune microenvironment in patients with respiratory allergic diseases. Functional enrichment analysis suggested that glutathione derivative metabolic processes, calcium signaling pathways, and regulation of autophagy may be potential targets for m6A regulation in the pathogenesis of respiratory allergies. Immune microenvironment infiltration analysis revealed a correlation between the expression of key m6A regulators and immune cell infiltration. Finally, we propose that the possible application of topical nasal methylprednisolone for the treatment of respiratory allergies is related to the regulation of *METTL14*, providing new ways of investigating the treatment of respiratory allergies.

## Supplementary Information

**Additional file 1**: **Table S1.** The results of PPI network of 4 hub m6A regulators.

**Additional file 2**: **Table S2.** The results of GO analysis of the 4 hub m6A regulators.

**Additional file 3**: **Table S3.** The results of GSEA analysis of the 4 hub m6A regulators.

## Data Availability

Publicly available datasets were analyzed in this study. The dataset GSE46171 can be found here: Gene Expression Omnibus(GEO) database (https://www.ncbi.nlm.nih.gov/geo/).
