# Supplementary material for: Microenvironment modulation by key regulators of RNA N6-methyladenosine modification in respiratory allergic diseases
Source: BMC Pulm Med. 2023 Jun 16;23:210. doi: 10.1186/s12890-023-02499-0 (PMC10276419; doi:10.1186/s12890-023-02499-0)
Supplement: Supplementary file 1 — Additional file 1: Table S1. The results of PPI network of 4 hub m6A regulators. [file 12890_2023_2499_MOESM1_ESM.doc]

Table S1: The results of PPI network of 4 hub m6A regulators.

| GENE | PROTEIN |
| --- | --- |
| METTL14 | ENO1 |
|  | CDC7 |
|  | TMEM97 |
|  | CCNI |
|  | USP22 |
|  | TPT1 |
|  | SRSF1 |
|  | SH3GL1 |
|  | PLK4 |
|  | PRKACB |
|  | TPM3 |
|  | MCM2 |
|  | PPP6R1 |
|  | YWHAQ |
|  | MTCH1 |
|  | DBT |
|  | SERF2 |
|  | CLIC4 |
|  | RALGAPA1 |
|  | TMEM64 |
|  | HIBCH |
|  | SNX2 |
|  | ITSN1 |
|  | CCNL2 |
|  | YWHAZ |
|  | MICU2 |
|  | FAM84B |
|  | SUMO3 |
|  | NAP1L3 |
|  | KAZN |
|  | TEAD4 |
|  | AC015813.2 |
| RBM15 | ZZEF1 |
|  | GLG1 |
|  | TTLL12 |
|  | CAB39L |
|  | MAZ |
|  | EFTUD2 |
|  | MDK |
|  | KLHL12 |
|  | CALD1 |
|  | RLIM |
|  | RAN |
|  | HMGA1 |
|  | ALDH16A1 |
|  | WDR1 |
|  | PCM1 |
|  | CD82 |
|  | SMIM24 |
|  | DDX17 |
|  | SUN2 |
|  | TM9SF4 |
|  | GPI |
|  | NAMPT |
|  | CASP2 |
|  | SRSF3 |
|  | EIF4G1 |
|  | POLE4 |
|  | IGFBP2 |
|  | AMOT |
|  | IRF3 |
|  | NARS |
|  | CERS2 |
|  | SLC25A26 |
|  | PTGES2 |
|  | EIF4EBP2 |
|  | SLC16A1 |
|  | FBRS |
|  | ATAD3B |
|  | AGPAT3 |
|  | SQSTM1 |
|  | USP1 |
|  | RPL27A |
|  | PCDH7 |
|  | XXYLT1 |
|  | CLDN6 |
|  | GTF2E2 |
|  | ZNF841 |
|  | PPP1R2B |
|  | STRBP |
|  | SLC25A6 |
|  | JAKMIP2 |
|  | OAZ2 |
|  | AIDA |
|  | H2AFX |
|  | RPL14 |
|  | ENTPD6 |
|  | ATL1 |
|  | RACK1 |
| RBM15B | ARF5 |
|  | PTBP1 |
|  | SLC38A5 |
|  | RALBP1 |
|  | CD74 |
|  | RNF10 |
|  | ALAS1 |
|  | PHF20 |
|  | USP28 |
|  | PKP2 |
|  | WNK1 |
|  | ISOC2 |
|  | ADGRL1 |
|  | HMMR |
|  | SEMA3A |
|  | MAP2K7 |
|  | SMAP2 |
|  | GNAS |
|  | GLG1 |
|  | SCFD1 |
|  | ARCN1 |
|  | ACOT7 |
|  | HNRNPM |
|  | TSPO |
|  | RPL3 |
|  | CERK |
|  | AHCY |
|  | MAN2B1 |
|  | SLC1A5 |
|  | RPL18A |
|  | ABCA2 |
|  | HOXB6 |
|  | MLX |
|  | PPP1R9B |
|  | CBL |
|  | MDK |
|  | KCTD10 |
|  | ACAD10 |
|  | TPI1 |
|  | QKI |
|  | GOLPH3 |
|  | EIF4G1 |
|  | TTL |
|  | NRBP1 |
|  | MAD2L2 |
|  | SLC2A1 |
|  | RCOR3 |
|  | SET |
|  | EPCAM |
|  | SNX19 |
|  | HOXA7 |
|  | DEK |
|  | GRPR |
|  | KTN1 |
|  | ATF4 |
|  | DDA1 |
|  | RTN4IP1 |
|  | LSP1 |
|  | EIF2S3 |
|  | SESN2 |
|  | UBE2D2 |
|  | PPP1R1B |
|  | LLGL1 |
|  | LIN28A |
|  | ILKAP |
|  | ANKRD17 |
|  | EIF5A |
|  | PRMT7 |
|  | TPT1 |
|  | BTG1 |
|  | CAPRIN1 |
|  | CCDC142 |
|  | FHOD1 |
|  | TBC1D4 |
|  | SCRN1 |
|  | DNAJC1 |
|  | TUBB2A |
|  | SMAD6 |
|  | SAE1 |
|  | FBXL17 |
|  | TMEM209 |
|  | EBP |
|  | SESN3 |
|  | MZT2B |
|  | FMN2 |
|  | MCU |
|  | BACH1 |
|  | ANKRD9 |
|  | EEF1A1 |
|  | SASS6 |
|  | ST3GAL2 |
|  | SKI |
|  | ALDH4A1 |
|  | LSS |
|  | INAVA |
|  | LRRC58 |
|  | TKT |
|  | CMBL |
|  | CITED2 |
|  | BRI3 |
|  | PSIP1 |
|  | ZHX1 |
|  | SNAPC4 |
|  | RPL13 |
|  | KMT2D |
|  | HIST1H1E |
|  | POLR3D |
|  | NSG1 |
|  | GPS1 |
|  | NIPA1 |
|  | PGAM1 |
|  | CTPS1 |
|  | RPS21 |
|  | CFL1 |
|  | NAA16 |
|  | TIMM22 |
|  | FBXO34 |
|  | MXRA7 |
|  | TMEM50A |
|  | COPB2 |
|  | PMEL |
|  | CCDC84 |
|  | PTMA |
|  | SAMD11 |
|  | FANCA |
|  | JPT1 |
|  | LONP1 |
|  | XRCC6 |
|  | STMN3 |
|  | ZNF770 |
|  | APRT |
|  | RACK1 |
|  | TRIM27 |
|  | MRPL53 |
|  | ZBTB10 |
|  | PI4KA |
|  | KCTD7 |
|  | TTC4 |
|  | RBM12 |
|  | CUX1 |
|  | MAPK9 |
|  | ANAPC4 |
|  | EPN1 |
|  | ROCK1 |
|  | PABPC1 |
|  | JADE1 |
|  | PPP2R5C |
|  | CBFA2T2 |
|  | XRCC5 |
|  | EPB41L3 |
|  | NFE2L1 |
|  | STARD7 |
|  | SF3B2 |
|  | CRKL |
|  | SNU13 |
|  | DEPDC5 |
|  | TIMM9 |
|  | NFKBIA |
|  | TPD52L2 |
|  | CSK |
|  | CLPTM1 |
|  | EZH2 |
|  | PFN1 |
|  | SUPT6H |
|  | PHF12 |
|  | WFS1 |
|  | GAPDH |
|  | HECA |
|  | TNFSF4 |
|  | RCN2 |
|  | CAMSAP2 |
|  | NPC2 |
|  | PDE1B |
|  | HOXC13 |
|  | MATN4 |
|  | STAU1 |
|  | SH2D3A |
|  | SYMPK |
|  | SNRPB |
|  | INTS11 |
|  | PODXL |
|  | LOXL1 |
|  | SPCS3 |
|  | APOE |
|  | OLFM1 |
|  | DKC1 |
|  | TUBG1 |
|  | RAN |
|  | H3F3B |
|  | MPRIP |
|  | BTBD2 |
|  | RPS15A |
|  | GDF11 |
|  | KRT7 |
|  | HNRNPA1 |
|  | RC3H1 |
|  | SRSF1 |
|  | SKIL |
|  | RPS6 |
|  | RPLP1 |
|  | HNRNPD |
|  | SINHCAF |
|  | ETFA |
|  | ST8SIA2 |
|  | PTRH2 |
|  | VASH2 |
|  | RASGEF1C |
|  | RPL7A |
|  | TNKS1BP1 |
|  | FAU |
|  | FOXO1 |
|  | DIP2C |
|  | RNF144A |
|  | SAV1 |
|  | ZFP36L2 |
|  | HNRNPU |
|  | ZNF689 |
|  | TMED4 |
|  | AMFR |
|  | CALM3 |
|  | PKNOX1 |
|  | SRSF2 |
|  | RAVER2 |
|  | FSTL1 |
|  | ZDHHC3 |
|  | ZNF589 |
|  | TAF1D |
|  | CEP57 |
|  | HSP90B1 |
|  | NUDT21 |
|  | TUBA1A |
|  | EEF2 |
|  | CDK2AP2 |
|  | MLST8 |
|  | ECI1 |
|  | DDB1 |
|  | UBTD2 |
|  | SLC25A6 |
|  | PRELID1 |
|  | ZNF282 |
|  | CDK1 |
|  | SEMA3E |
|  | PPP1CA |
|  | CD19 |
|  | SLC25A22 |
|  | HNRNPA0 |
|  | CTXN1 |
|  | ZNRF2 |
|  | ALYREF |
|  | CLDN6 |
|  | FAF1 |
|  | ZNRF1 |
|  | KRT10 |
|  | TPCN1 |
|  | TUBB |
|  | SIAH1 |
|  | C6orf106 |
|  | PDGFA |
|  | TAP2 |
|  | MZT1 |
|  | ANKRD28 |
|  | ARL2 |
|  | CSNK1E |
|  | ZSWIM8 |
|  | CASTOR3 |
|  | EIF6 |
|  | AL669918.1 |
|  | MTRNR2L1 |
|  | AC138811.2 |
|  | RCC1L |
|  | PSMB3 |
|  | Z82190.2 |
|  | AC139530.2 |
|  | MRPL12 |
|  | BAHCC1 |
|  | AC010422.5 |
|  | MROH7-TTC4 |
|  | AC027644.4 |
| METTL16 | CALCOCO1 |
|  | CD44 |
|  | PNPLA6 |
|  | LNX1 |
|  | DOCK9 |
|  | HNRNPC |
|  | EIF3L |
|  | USP14 |
|  | SFRP1 |
|  | C19orf44 |
|  | FBL |
|  | HPN |
|  | PTGES3 |
|  | CYTIP |
|  | GORASP2 |
|  | AP2B1 |
|  | ZNF263 |
|  | STARD7 |
|  | TMED2 |
|  | RTRAF |
|  | RPLP0 |
|  | ITGA6 |
|  | ZFHX4 |
|  | AGO1 |
|  | AAAS |
|  | PPM1F |
|  | DDX17 |
|  | YY1 |
|  | CLIP2 |
|  | DHCR24 |
|  | TMEM214 |
|  | SOHLH2 |
|  | TUBA1B |
|  | PLA2G12A |
|  | SAT1 |
|  | TULP4 |
|  | PLXNA3 |
|  | RPL27 |
|  | LIN28A |
|  | EIF5A |
|  | RFC3 |
|  | KIF23 |
|  | RPS24 |
|  | MAN2C1 |
|  | ERBB2 |
|  | PRCC |
|  | GNA12 |
|  | ALDOA |
|  | EEF1A1 |
|  | NPTN |
|  | SLC35B2 |
|  | EPB41 |
|  | ALDH4A1 |
|  | GNE |
|  | C21orf58 |
|  | SAFB |
|  | SGCB |
|  | CGGBP1 |
|  | VCP |
|  | HSP90B1 |
|  | HDHD2 |
|  | RPS23 |
|  | ZNF793 |
|  | ZNF772 |
|  | ZNF649 |
|  | HLA-C |
|  | HLA-DPB1 |
|  | TAPBP |
|  | CCDC169-SOHLH2 |
|  | NACA2 |
|  | AC012254.2 |
|  | TAF9 |
|  | PIP4K2B |
|  | PIMREG |
|  | SDF2 |
|  | FBXO18 |
|  | PGD |
|  | CNOT9 |
|  | NDUFB11 |
|  | GLUD1 |
|  | ITGB1 |
|  | ANAPC1 |
|  | SKA1 |
|  | TMEM164 |
|  | ADAR |
|  | RFT1 |
|  | NDUFB8 |
|  | EEF2 |
|  | TK1 |
|  | HNRNPF |
|  | NPM1 |
|  | TBX1 |
|  | DDX51 |
|  | TMLHE |
|  | EXD3 |
|  | LIN28B |
|  | NIF3L1 |
|  | ZNF587 |
|  | MT-CO2 |
|  | MT-ND4 |
|  | SDHD |
|  | SARNP |
|  | AL133352.1 |
|  | AC023055.1 |
|  | AC008878.2 |
|  | AC096887.1 |
