# Supplementary material for: Microenvironment modulation by key regulators of RNA N6-methyladenosine modification in respiratory allergic diseases
Source: BMC Pulm Med. 2023 Jun 16;23:210. doi: 10.1186/s12890-023-02499-0 (PMC10276419; doi:10.1186/s12890-023-02499-0)
Supplement: Supplementary file 2 — Additional file 2: Table S2. The results of GO analysis of the 4 hub m6A regulators. [file 12890_2023_2499_MOESM2_ESM.doc]

Table S2: The results of GO analysis of the 4 hub m6A regulators.

| ONTOLOGY | Description | p.adjust | Count |
| --- | --- | --- | --- |
| BP | RNA methylation | 3.32E-08 | 4 |
| BP | RNA modification | 2.83E-07 | 4 |
| BP | macromolecule methylation | 2.18E-06 | 4 |
| BP | regulation of mRNA metabolic process | 2.18E-06 | 4 |
| BP | methylation | 2.18E-06 | 4 |
| BP | RNA splicing, via transesterification reactions with bulged adenosine as nucleophile | 2.18E-06 | 4 |
| BP | mRNA splicing, via spliceosome | 2.18E-06 | 4 |
| BP | RNA splicing, via transesterification reactions | 2.18E-06 | 4 |
| BP | RNA splicing | 4.41E-06 | 4 |
| BP | regulation of mRNA splicing, via spliceosome | 5.64E-06 | 3 |
| BP | regulation of RNA splicing | 1.29E-05 | 3 |
| BP | regulation of mRNA processing | 1.29E-05 | 3 |
| CC | methyltransferase complex | 2.92E-06 | 3 |
| CC | nuclear speck | 4.28E-03 | 2 |
| CC | nuclear envelope | 4.28E-03 | 2 |
| MF | mRNA methyltransferase activity | 2.53E-05 | 2 |
| MF | RNA methyltransferase activity | 4.91E-04 | 2 |
| MF | S-adenosylmethionine-dependent methyltransferase activity | 1.81E-03 | 2 |
| MF | methyltransferase activity | 2.30E-03 | 2 |
| MF | transferase activity, transferring one-carbon groups | 2.30E-03 | 2 |
| MF | catalytic activity, acting on RNA | 5.03E-03 | 2 |
