# Supplementary material for: Microenvironment modulation by key regulators of RNA N6-methyladenosine modification in respiratory allergic diseases
Source: BMC Pulm Med. 2023 Jun 16;23:210. doi: 10.1186/s12890-023-02499-0 (PMC10276419; doi:10.1186/s12890-023-02499-0)
Supplement: Supplementary file 3 — Additional file 3: Table S3. The results of GSEA analysis of the 4 hub m6A regulators. [file 12890_2023_2499_MOESM3_ESM.doc]

Table S3: The results of GSEA analysis of the 4 hub m6A regulators.

| **GENE** | **DESCRIPTION** | **ENRICHMENT SCORE** | **NES** | **NOM p-val** |
| --- | --- | --- | --- | --- |
| METTL14 | GOBP-Glutathione derivative metabolic process | -0.62 | -1.52 | 4.57E-02 |
|  | GOBP-Glutathione metabolic process | -0.42 | -1.46 | 2.73E-02 |
|  | GOBP-Histone methylation | 0.42 | 1.74 | 0.00E+00 |
|  | GOBP-Intrinsic apoptotic signaling pathway in response to dna damage by p53 class mediator | 0.49 | 1.78 | 4.13E-03 |
|  | GOBP-Peptidyl lysine methylation | 0.40 | 1.70 | 4.17E-03 |
|  | GOBP-Peptidyl lysine trimethylation | 0.54 | 1.77 | 2.18E-03 |
|  | GOBP-Plasma membrane fusion | -0.57 | -1.39 | 4.85E-02 |
|  | GOBP-Positive regulation of calcium ion transmembrane transporter activity | -0.52 | -1.40 | 3.67E-02 |
|  | GOBP-Positive regulation of protein modification by small protein conjugation or removal | 0.42 | 1.67 | 0.00E+00 |
|  | GOBP-Protein methylation | 0.38 | 1.70 | 0.00E+00 |
|  | GOBP-Regulation of histone deacetylation | 0.53 | 1.77 | 6.10E-03 |
|  | GOBP-Regulation of histone methylation | 0.54 | 1.79 | 2.11E-03 |
|  | GOBP-Regulation of histone modification | 0.42 | 1.64 | 2.05E-03 |
|  | GOBP-rRNA catabolic process | -0.44 | -1.52 | 4.38E-02 |
|  | GOCC-Pml body | 0.41 | 1.72 | 4.25E-03 |
|  | GOCC-Transcription repressor complex | 0.42 | 1.68 | 2.10E-03 |
|  | GOMF-Carbohydrate cation symporter activity | -0.60 | -1.38 | 4.95E-02 |
|  | GOMF-GDP binding | 0.46 | 1.66 | 4.12E-03 |
|  | GOMF-Glutathione peroxidase activity | -0.54 | -1.50 | 2.75E-02 |
|  | KEGG-Endometrial cancer | 0.44 | 1.49 | 5.74E-02 |
|  | KEGG-GAP junction | 0.37 | 1.35 | 4.58E-02 |
|  | KEGG-Glutathione metabolism | -0.43 | -1.42 | 6.24E-02 |
|  | KEGG-Oxidative phosphorylation | -0.30 | -1.30 | 2.26E-01 |
|  | KEGG-Parkinsons disease | -0.27 | -1.26 | 2.35E-01 |
|  | KEGG-Pentose phosphate pathway | -0.47 | -1.41 | 7.78E-02 |
|  | KEGG-Prostate cancer | 0.41 | 1.46 | 3.81E-02 |
|  | KEGG-Ribosome | -0.42 | -1.33 | 1.87E-01 |
|  | KEGG-Spliceosome | 0.30 | 1.49 | 9.36E-02 |
|  | KEGG-Ubiquitin mediated proteolysis | 0.27 | 1.45 | 2.50E-02 |
| METTL16 | GOBP-2 Oxoglutarate metabolic process | 0.62 | 1.44 | 8.59E-02 |
|  | GOBP-Anoikis | -0.52 | -1.50 | 4.11E-02 |
|  | GOBP-Cell surface receptor signaling pathway involved in heart development | -0.59 | -1.48 | 3.99E-02 |
|  | GOBP-Cellular response to low density lipoprotein particle stimulus | -0.63 | -1.48 | 2.13E-02 |
|  | GOBP-Cholesterol storage | -0.58 | -1.48 | 3.21E-02 |
|  | GOBP-Circadian rhythm | -0.33 | -1.32 | 4.17E-02 |
|  | GOBP-Golgi to plasma membrane protein transport | 0.37 | 1.44 | 7.53E-02 |
|  | GOBP-Histone h4 k16 acetylation | -0.60 | -1.60 | 1.77E-02 |
|  | GOBP-Inactivation of mapk activity | -0.58 | -1.47 | 2.91E-02 |
|  | GOBP-Male meiosis i | -0.63 | -1.55 | 1.99E-02 |
|  | GOBP-Melanocyte differentiation | -0.66 | -1.49 | 2.18E-02 |
|  | GOBP-Negative regulation of epithelial cell differentiation | -0.63 | -1.49 | 2.06E-02 |
|  | GOBP-Negative regulation of fatty acid metabolic process | -0.50 | -1.43 | 3.37E-02 |
|  | GOBP-Nucleus organization | -0.31 | -1.41 | 3.66E-02 |
|  | GOBP-Pigment cell differentiation | -0.61 | -1.51 | 3.33E-02 |
|  | GOBP-Positive regulation of lipid storage | -0.59 | -1.57 | 2.95E-02 |
|  | GOBP-Positive regulation of steroid metabolic process | -0.60 | -1.41 | 3.38E-02 |
|  | GOBP-Protein autoprocessing | -0.62 | -1.56 | 1.34E-02 |
|  | GOBP-Protein transmembrane import into intracellular organelle | 0.41 | 1.45 | 1.25E-01 |
|  | GOBP-Response to gonadotropin | -0.63 | -1.51 | 1.15E-02 |
|  | GOBP-Sodium ion homeostasis | -0.54 | -1.43 | 4.41E-02 |
|  | GOBP-Vasodilation | -0.59 | -1.48 | 2.52E-02 |
|  | GOBP-Ventricular system development | 0.66 | 1.54 | 5.17E-02 |
|  | GOCC-Inner mitochondrial membrane protein complex | 0.38 | 1.43 | 1.42E-01 |
|  | GOCC-Mitochondrial protein containing complex | 0.34 | 1.44 | 1.87E-01 |
|  | GOCC-NADH dehydrogenase complex | 0.51 | 1.45 | 6.73E-02 |
|  | GOCC-Respirasome | 0.42 | 1.44 | 1.36E-01 |
|  | GOCC-Respiratory chain complex | 0.43 | 1.44 | 1.20E-01 |
|  | GOMF-14-3-3 Protein binding | -0.51 | -1.61 | 2.93E-02 |
|  | GOMF-Histone methyltransferase activity | -0.40 | -1.55 | 2.20E-02 |
|  | GOMF-Scaffold protein binding | -0.52 | -1.58 | 5.85E-03 |
|  | GOMF-Sodium independent organic anion transmembrane transporter activity | -0.69 | -1.49 | 2.24E-02 |
|  | GOMF-Translation initiation factor binding | -0.50 | -1.56 | 4.15E-02 |
|  | KEGG-Adipocytokine signaling pathway | -0.48 | -1.47 | 4.87E-02 |
|  | KEGG-Base excision repair | -0.44 | -1.41 | 1.49E-01 |
|  | KEGG-Endometrial cancer | -0.42 | -1.50 | 5.69E-02 |
|  | KEGG-Glioma | -0.47 | -1.46 | 4.97E-02 |
|  | KEGG-Non small cell lung cancer | -0.46 | -1.48 | 5.85E-02 |
|  | KEGG-Oxidative phosphorylation | 0.41 | 1.63 | 5.39E-02 |
|  | KEGG-arkinsons disease | 0.31 | 1.40 | 1.52E-01 |
|  | KEGG-Pentose phosphate pathway | 0.45 | 1.37 | 1.29E-01 |
|  | KEGG-Ribosome | 0.48 | 1.38 | 1.60E-01 |
|  | KEGG-RNA polymerase | 0.39 | 1.36 | 1.48E-01 |
|  | KEGG-type II diabetes mellitus | -0.58 | -1.42 | 5.21E-02 |
| RBM15 | GOBP-ARP2-3 complex mediated actin nucleation | 0.44 | 1.66 | 2.50E-02 |
|  | GOBP-Aspartate family amino acid biosynthetic process | 0.63 | 1.79 | 1.97E-03 |
|  | GOBP-Blood coagulation intrinsic pathway | -0.64 | -1.56 | 1.55E-02 |
|  | GOBP-Chaperone mediated protein complex assembly | -0.50 | -1.61 | 3.20E-02 |
|  | GOBP-Cotranslational protein targeting to membrane | 0.47 | 1.74 | 3.01E-02 |
|  | GOBP-Cyclic nucleotide biosynthetic process | -0.62 | -1.55 | 1.17E-02 |
|  | GOBP-Establishment of protein localization to endoplasmic reticulum | 0.46 | 1.89 | 1.44E-02 |
|  | GOBP-Establishment of protein localization to membrane | 0.26 | 1.68 | 0.00E+00 |
|  | GOBP-Golgi to plasma membrane transport | 0.37 | 1.66 | 1.78E-02 |
|  | GOBP-Microvillus organization | -0.61 | -1.56 | 8.15E-03 |
|  | GOBP-NADPH regeneration | 0.54 | 1.66 | 2.04E-02 |
|  | GOBP-Negative regulation of translational initiation | -0.47 | -1.54 | 3.27E-02 |
|  | GOBP-Nuclear transcribed mrna catabolic process | 0.26 | 1.71 | 3.82E-02 |
|  | GOBP-Protein k48 linked ubiquitination | 0.44 | 1.78 | 8.06E-03 |
|  | GOBP-Protein localization to endoplasmic reticulum | 0.41 | 1.94 | 1.65E-02 |
|  | GOBP-Regulation of oxidative phosphorylation | 0.54 | 1.65 | 8.21E-03 |
|  | GOBP-RNA polyadenylation | -0.39 | -1.56 | 2.59E-02 |
|  | GOBP Sulfur amino acid biosynthetic process | 0.55 | 1.67 | 1.96E-02 |
|  | GOBP-Synaptic transmission glutamatergic | -0.57 | -1.60 | 0.00E+00 |
|  | GOBP-Translational initiation | 0.33 | 1.85 | 2.01E-02 |
|  | GOCC-AMPA glutamate receptor complex | -0.66 | -1.53 | 1.98E-02 |
|  | GOCC-Intrinsic component of mitochondrial outer membrane | 0.58 | 1.68 | 3.92E-03 |
|  | GOCC-Mediator complex | 0.47 | 1.67 | 2.04E-03 |
|  | GOCC-mRNA cleavage and polyadenylation specificity factor complex | -0.59 | -1.73 | 4.02E-03 |
|  | GOCC-mRNA cleavage factor complex | -0.54 | -1.78 | 6.05E-03 |
|  | GOMF-G Protein coupled amine receptor activity | -0.59 | -1.55 | 6.12E-03 |
|  | GOMF-Protein transmembRANE Transporter activity | 0.59 | 1.67 | 1.57E-02 |
|  | GOMF-Ubiquitin like protein conjugating enzyme activity | 0.55 | 1.68 | 2.41E-02 |
|  | KEGG-Adherens junction | 0.48 | 1.72 | 3.84E-03 |
|  | KEGG-Alzheimers disease | 0.28 | 1.40 | 6.20E-02 |
|  | KEGG-Calcium signaling pathway | -0.36 | -1.29 | 4.47E-02 |
|  | KEGG-Ether lipid metabolism | -0.49 | -1.37 | 6.68E-02 |
|  | KEGG-Galactose metabolism | -0.52 | -1.45 | 7.66E-02 |
|  | KEGG-Glycolysis gluconeogenesis | -0.36 | -1.38 | 6.02E-02 |
|  | KEGG-Other glycan degradation | -0.47 | -1.37 | 1.16E-01 |
|  | KEGG-Parkinsons disease | 0.38 | 1.69 | 3.87E-02 |
|  | KEGG-Regulation of autophagy | -0.50 | -1.40 | 1.22E-01 |
|  | KEGG-Ribosome | 0.53 | 1.58 | 3.93E-02 |
|  | KEGG-Ubiquitin mediated proteolysis | 0.30 | 1.59 | 7.91E-03 |
| RBM15B | GOBP-Cell differentiation involved in embryonic placenta development | 0.72 | 1.55 | 1.40E-02 |
|  | GOBP-Glutathione derivative metabolic process | -0.70 | -1.49 | 4.04E-02 |
|  | GOBP-Intrinsic apoptotic signaling pathway by p53 class mediator | 0.41 | 1.58 | 1.36E-02 |
|  | GOBP-Intrinsic apoptotic signaling pathway in response to dna damage by p53 class mediator | 0.46 | 1.65 | 1.39E-02 |
|  | GOBP-Negative regulation of calcium ion transmembrane transporter activity | -0.51 | -1.47 | 3.30E-02 |
|  | GOBP-Negative regulation of calcium ion transport into cytosol | -0.49 | -1.43 | 3.59E-02 |
|  | GOBP-Negative regulation of chromatin organization | 0.50 | 1.56 | 1.40E-02 |
|  | GOBP-Negative regulation of histone modification | 0.49 | 1.58 | 1.39E-02 |
|  | GOBP-Outer dynein arm assembly | -0.91 | -1.35 | 2.36E-02 |
|  | GOBP-Positive regulation of calcium ion transmembrane transporter activity | -0.55 | -1.52 | 9.84E-03 |
|  | GOBP-Regulation of calcium ion transmembrane transporter activity | -0.43 | -1.39 | 1.55E-02 |
|  | GOBP-Regulation of cardiac muscle contraction by calcium ion signaling | -0.48 | -1.41 | 3.06E-02 |
|  | GOBP-Regulation of chromatin organization | 0.44 | 1.59 | 1.39E-02 |
|  | GOBP-Regulation of dna templated transcription elongation | 0.51 | 1.70 | 7.89E-03 |
|  | GOBP-Regulation of histone methylation | 0.54 | 1.67 | 3.97E-03 |
|  | GOBP-Regulation of histone modification | 0.44 | 1.58 | 8.03E-03 |
|  | GOBP-Regulation of protein polyubiquitination | 0.60 | 1.63 | 1.00E-02 |
|  | GOBP-Regulation of ryanodine sensitive calcium release channel activity | -0.56 | -1.51 | 7.69E-03 |
|  | GOBP-Regulation of transcription elongation from rna polymerase ii promoter | 0.61 | 1.63 | 1.41E-02 |
|  | GOBP-Response to antibiotic | 0.49 | 1.55 | 1.20E-02 |
|  | GOBP-Response to gonadotropin | 0.67 | 1.58 | 9.90E-03 |
|  | GOBP-Response to ionizing radiation | 0.52 | 1.59 | 6.02E-03 |
|  | GOCC-9plus2 motile cilium | -0.72 | -1.51 | 3.64E-02 |
|  | GOCC-Protein kinase complex | 0.42 | 1.56 | 1.17E-02 |
|  | GOCC-Sex chromosome | 0.73 | 1.63 | 7.72E-03 |
|  | GOCC-Sperm midpiece | -0.58 | -1.51 | 3.82E-02 |
|  | GOMF-ATP dependent microtubule motor activity minus end directed | -0.89 | -1.38 | 4.04E-02 |
|  | GOMF-Atpase coupled intramembrane lipid transporter activity | 0.64 | 1.63 | 3.84E-03 |
|  | GOMF-Carbohydrate cation symporter activity | -0.74 | -1.74 | 0.00E+00 |
|  | GOMF-Glutathione peroxidase activity | -0.60 | -1.59 | 2.73E-02 |
|  | KEGG-Bladder cancer | 0.59 | 1.46 | 4.64E-02 |
|  | KEGG-Colorectal cancer | 0.42 | 1.46 | 3.97E-02 |
|  | KEGG-Glioma | 0.51 | 1.48 | 2.77E-02 |
|  | KEGG-Glutathione metabolism | -0.43 | -1.40 | 9.31E-02 |
|  | KEGG-Oxidative phosphorylation | -0.31 | -1.28 | 2.53E-01 |
|  | KEGG-P53 signaling pathway | 0.54 | 1.52 | 2.62E-02 |
|  | KEGG-Pancreatic cancer | 0.48 | 1.45 | 4.86E-02 |
|  | KEGG-Pathways in cancer | 0.39 | 1.39 | 3.14E-02 |
|  | KEGG-Pentose phosphate pathway | -0.48 | -1.41 | 1.09E-01 |
|  | KEGG-RNA polymerase | -0.33 | -1.23 | 2.30E-01 |
|  | KEGG-Steroid biosynthesis | -0.49 | -1.26 | 2.63E-01 |
|  | KEGG-Ubiquitin mediated proteolysis | 0.32 | 1.57 | 1.04E-02 |
